# Cyp26 Enzymes Facilitate Second Heart Field Progenitor Addition and Maintenance of Ventricular Integrity

**DOI:** 10.1371/journal.pbio.2000504

**Published:** 2016-11-28

**Authors:** Ariel B. Rydeen, Joshua S. Waxman

**Affiliations:** 1 Molecular Cardiovascular Biology Division and Heart Institute, Cincinnati Children’s Hospital Medical Center, Cincinnati, Ohio, United States of America; 2 Molecular and Developmental Biology Graduate Program, University of Cincinnati Medical Center, Cincinnati, Ohio, United States of America; University of Pittsburgh, United States of America

## Abstract

Although retinoic acid (RA) teratogenicity has been investigated for decades, the mechanisms underlying RA-induced outflow tract (OFT) malformations are not understood. Here, we show zebrafish embryos deficient for Cyp26a1 and Cyp26c1 enzymes, which promote RA degradation, have OFT defects resulting from two mechanisms: first, a failure of second heart field (SHF) progenitors to join the OFT, instead contributing to the pharyngeal arch arteries (PAAs), and second, a loss of first heart field (FHF) ventricular cardiomyocytes due to disrupted cell polarity and extrusion from the heart tube. Molecularly, excess RA signaling negatively regulates *fibroblast growth factor 8a* (*fgf8a*) expression and positively regulates *matrix metalloproteinase 9* (*mmp9*) expression. Although restoring Fibroblast growth factor (FGF) signaling can partially rescue SHF addition in Cyp26 deficient embryos, attenuating matrix metalloproteinase (MMP) function can rescue both ventricular SHF addition and FHF integrity. These novel findings indicate a primary effect of RA-induced OFT defects is disruption of the extracellular environment, which compromises both SHF recruitment and FHF ventricular integrity.

## Author Summary

Retinoic acid (RA) is the most active metabolic product of vitamin A. The embryonic heart is particularly sensitive to inappropriate RA levels, with cardiac outflow tract (OFT) defects among the most common RA-induced malformations. However, the mechanisms underlying these RA-induced defects are not understood. Cyp26 enzymes facilitate degradation of RA and thus are required to limit RA levels in early development. Here, we present evidence that loss of Cyp26 enzymes induces cardiac OFT defects through two mechanisms. First, we find that Cyp26-deficient zebrafish embryos fail to add later-differentiating ventricular cardiac progenitors to the OFT, with some of these progenitors instead contributing to the nearby arch arteries. Second, Cyp26-deficient embryos cannot maintain the integrity of the nascent heart tube, with ventricular cells within the heart tube losing their polarity and being extruded. Our data indicate that excess expression of matrix metalloproteinase 9, an enzyme that degrades the extracellular matrix, underlies both the cardiac progenitor addition and heart tube integrity defects seen in Cyp26-deficient embryos. Our findings highlight perturbation of the extracellular matrix as a major cause of RA-induced cardiac OFT defects that specifically disrupt ventricular development at later stages than previously appreciated.

## Introduction

The heart is the first organ to develop and function in all vertebrates. Improper heart development can lead to congenital heart defects (CHDs), which impinge on normal embryogenesis and can result in embryonic or neonatal lethality [1,2]. Construction of a functional vertebrate heart requires the precisely coordinated development of two sources of cardiomyocyte progenitors [3]. Progenitors within the anterior lateral plate mesoderm give rise to early-differentiating cardiomyocytes of the first heart field (FHF), which generate the initial heart tube [3,4]. Progenitors within the adjacent, medial pharyngeal mesoderm give rise to later-differentiating cardiomyocytes of the second heart field (SHF), which augments the heart through accretion to both the arterial and venous poles [3,4]. There has been considerable effort in elucidating the mechanisms of vertebrate SHF development. Numerous studies examining mouse and zebrafish embryos have demonstrated defects in SHF development that result in outflow tract (OFT) defects [5–8]. Furthermore, there is a significant need to understand the mechanisms underlying OFT development, because, in humans, OFT defects comprise almost 30% of CHDs, which are the most common class of developmental malformations [2]. Despite advances in understanding many of the intricate signaling mechanisms that direct appropriate SHF development in vertebrates, the molecular etiologies underlying OFT defects still remain poorly understood.

It has long been established that proper levels of retinoic acid (RA), a metabolic product of vitamin A, are required for proper OFT development [9]. In particular, it is critical to limit embryonic RA levels, as excess RA is a potent teratogen [9–11]. The most common consequences of RA embryopathies are conotruncal and aortic arch malformations of the OFT [9,10]. The major mechanism limiting RA levels within vertebrate embryos are the Cyp26 enzymes (Cyp26a1, Cyp26b1, and Cyp26c1), which facilitate RA degradation [12,13]. Cyp26 enzyme deficiency occurs in several human developmental syndromes with OFT malformations, including DiGeorge Syndrome and Antley–Bixler Syndrome [14,15]. Previous studies in mice and zebrafish have shown loss of Cyp26a1 or both Cyp26a1 and Cyp26c1 results in heart malformations [16–19]. Although studies using exogenous RA treatment have indicated RA-induced OFT defects are in part due to the failure of cardiac neural crest addition to the arterial pole [20], excess RA from exogenous or endogenous sources likely also affects ventricular cardiomyocyte development in the OFT. Early exposure to high teratogenic levels of RA, which can inhibit ventricular progenitor specification during early patterning [21–23], may explain some ventricular OFT defects of the conus. However, the extraordinary sensitivity of ventricular OFT development to increased embryonic RA levels suggests more modest increases in RA levels at stages after early patterning may also produce OFT defects through unexplored mechanisms.

We recently demonstrated that Cyp26a1 and Cyp26c1 depletion (hereafter referred to as Cyp26 deficiency) causes an expansion of atrial progenitors at the expense of adjacent anterior vascular progenitors during early somitogenesis [16]. Despite these early patterning defects, development of the ventricular FHF within the nascent heart tube was not affected in Cyp26-deficient embryos [16]. Here, we show that Cyp26-deficient zebrafish embryos lack ventricular OFT development after the initial heart tube has formed. Interestingly, the ventricular OFT defects in Cyp26-deficient embryos are derived from two mechanisms: first, there is a failure of SHF progenitors to add to the nascent OFT, which instead contribute to the anterior PAAs, and second, there is a loss of cardiomyocyte polarity and extrusion of differentiated ventricular cardiomyocytes from the heart tube. Although FGF signaling is lost in Cyp26-deficient embryos, a lack of FGF signaling only partially accounts for the ventricular defects. Instead, we find that ventricular OFT defects in Cyp26-deficient embryos are predominantly caused by a parallel increase in *matrix metalloproteinase 9* (*mmp9*) expression. Thus, our data reveal a novel paradigm whereby the primary cause of ventricular OFT defects in Cyp26-deficient embryos is through matrix metalloproteinase (MMP)-induced disruption of the extracellular environment that impedes both SHF recruitment and the maintenance of FHF ventricular integrity.

## Results

### Cyp26-Deficient Embryos Have Decreased Ventricular Cardiomyocytes

**Fig 1:**
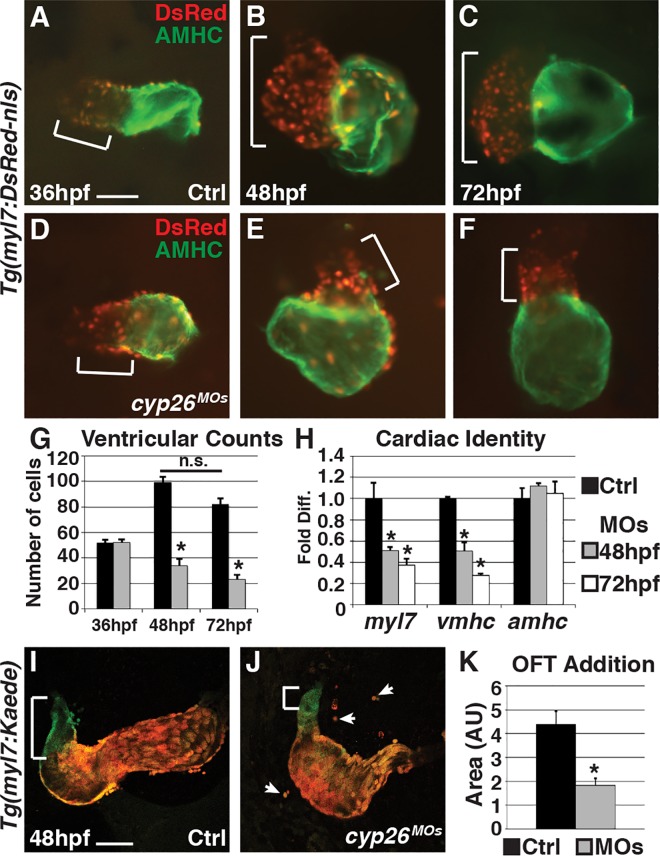
Cyp26-deficient embryos fail to add ventricular cardiomyocytes. (A–F) Control (Ctrl) and Cyp26-deficient *Tg*(*myl7*:*DsRed2-NLS*) embryo hearts at 36 (A,D), 48 (B,E), and 72 hpf (C,F). Ventricles are red only (brackets) and atria are green (AMHC immunostaining). (G) Graph indicating cardiomyocyte counts at 36 (*n* = 10 for control and *n* = 8 for Cyp26 deficient), 48 (*n* = 15 per group) and 72 hpf (*n* = 15 per group). (H) Graph indicating fold difference of mRNA relative to *β-actin* assayed with RT-qPCR for the cardiac differentiation markers *myl7*, *vmhc*, and *amhc*. (I,J) Confocal images of hearts from *Tg(myl7*:*Kaede)* embryos at 48 hpf that were photoconverted at 36 hpf. Brackets indicate ventricular addition (green only cells) to the hearts. Arrows denote cardiomyocytes outside of the heart tube. (K) Graph depicting quantification of the amount of ventricular addition to the OFT (*n* = 8 per group). Frontal views, anterior up (A–F); *n* > 20 embryos per group (A-F,I,J). Error bars are standard error of the mean (SEM), asterisks denote *p* < 0.05 compared to controls by Student’s *t* test. Scale bar: 50 μm.

Using previously validated morpholinos (MOs) [16,24], we found that Cyp26-deficient embryos display normal overt heart morphology and ventricular cardiomyocyte number at 36 hours post fertilization ([hpf], Fig 1A, 1D and 1G), confirming our previous observations [16]. However, after initial heart tube formation, Cyp26a1 and Cyp26c1 are both expressed in the pharyngeal arches adjacent to the OFT [25,26]. Additionally, analysis of *cyp26a1* expression, which is a well-characterized direct RA signaling target gene [27–31], and a transgenic RA sensor line [32] indicates Cyp26-deficient embryos have increased RA signaling at these later stages in the cardio-pharyngeal region (S1A–S1F Fig), suggesting that Cyp26 enzymes may also be required for proper heart development at later embryonic stages. Consistent with this hypothesis, examining Cyp26-deficient embryos at 48 and 72 hpf revealed hearts that become progressively more dysmorphic, correlating with a significant reduction of both ventricular cardiomyocyte number as well as expression of cardiac differentiation markers *myl7* (pan-cardiac) and *vmhc* (ventricular) using real-time quantitative polymerase chain reaction (RT-qPCR) (Fig 1B, 1C and 1E–1H). *Giraffe* (*gir*) mutants, which have a nonsense mutation in *cyp26a1* leading to a truncation prior to the catalytic domain [25], have more modest but similar aberrant heart morphology and loss of ventricular cardiomyocytes, a phenotype that is exacerbated when Cyp26c1 is concurrently depleted (referred to as *gir+c1*) (S2A–S2Q Fig). Treatment with the pan cytochrome P450 (Cyp) inhibitor Ketoconazole (Sigma) from the two-cell stage or exogenous RA from 24 hpf also produced hearts with similar morphology and reduction of ventricular cardiomyocyte number (S3A–S3F Fig). Additionally, treatment of Cyp26-deficient embryos with DEAB, an inhibitor of RA synthesis, or co-injection of *cyp26a1* mRNA with the *cyp26* MOs can restore heart morphology and cardiomyocyte number (S3G–S3P Fig). Importantly, the decrease in ventricular cells is not from an increased production of atrial cells. *Gir* mutants and embryos with Cyp26 knockdown using lower doses of MOs than were used to produce overt early patterning defects have a reduction in ventricular cardiomyocytes independent of any change in atrial cardiomyocyte number (S2R and S2S Fig) or the atrial differentiation marker *atrial myosin heavy chain* (*amhc*) (Fig 1H). Therefore, Cyp26 deficiency causes a specific loss of ventricular cardiomyocytes after the formation of the FHF-derived heart tube.

### SHF Progenitor Addition Is Disrupted in Cyp26 Deficient Embryos

A significant accretion of SHF-derived ventricular cardiomyocytes is observed in the hearts of control embryos after the generation of the predominantly FHF-derived heart tube between 36 and 48 hpf [5,33], which is not observed in Cyp26-deficient embryos (Fig 1G). To explicitly determine if SHF-derived ventricular cardiomyocytes fail to add in Cyp26-deficient embryos, we used the *Tg(myl7*:*Kaede)* line that facilitates analysis of cardiomyocyte addition through photoconversion of Kaede from green to red in differentiated cardiomyocytes [33]. Embryos were photoconverted just prior to 36 hpf and then imaged at 48 hpf, followed by quantification of ventricular addition (green only cells) using ImageJ software. We found that Cyp26-deficient, *gir*, and *gir+c1* embryos all have a significant decrease in the addition of later-differentiating ventricular cardiomyocytes to the OFT compared to control siblings (Fig 1I–1K and S2T–S2X Fig), confirming that SHF-derived ventricular cardiomyocyte addition is disrupted. To determine if other SHF derivatives are also lost, we examined *elastin b (elnb)*, which marks the SHF-derived smooth muscle of the bulbous arteriosus [34], using in situ hybridization (ISH). In Cyp26-deficient, *gir*, and *gir+c1* embryos, *elnb* expression was significantly diminished or abolished (S4A–S4D, S4E and S4G Fig). Together, these data confirm there is a failure of later-differentiating SHF derivatives to add to the OFT in Cyp26-deficient embryos.

We reasoned that the failure to add SHF-derived ventricular cardiomyocytes to the OFT in Cyp26-deficient embryos could be from either lack of SHF progenitor specification or an inability of SHF progenitors to properly join the extending heart tube. Because our previous work found that patterning of the anterior lateral plate mesoderm can be disrupted in Cyp26-deficient embryos [16], we first examined SHF progenitor specification. Using ISH and RT-qPCR, we did not find a loss in expression of the SHF progenitor markers *mef2cb* and *ltbp3*, or *nkx2*.*5*, which is expressed in cardiomyocyte and pharyngeal arch artery (PAA) progenitors [35], in Cyp26-deficient embryos compared to control siblings (S5A–S5K Fig). Counting *nkx2*.*5+* cells lateral to the cardiac cone in the cardio-pharyngeal region at 24 hpf also revealed that there is not a significant change in their number (S5L Fig). Thus, the loss of SHF-derived ventricular cardiomyocytes in Cyp26-deficient embryos is not due to early patterning defects that eliminate SHF progenitor specification.

**Fig 2:**
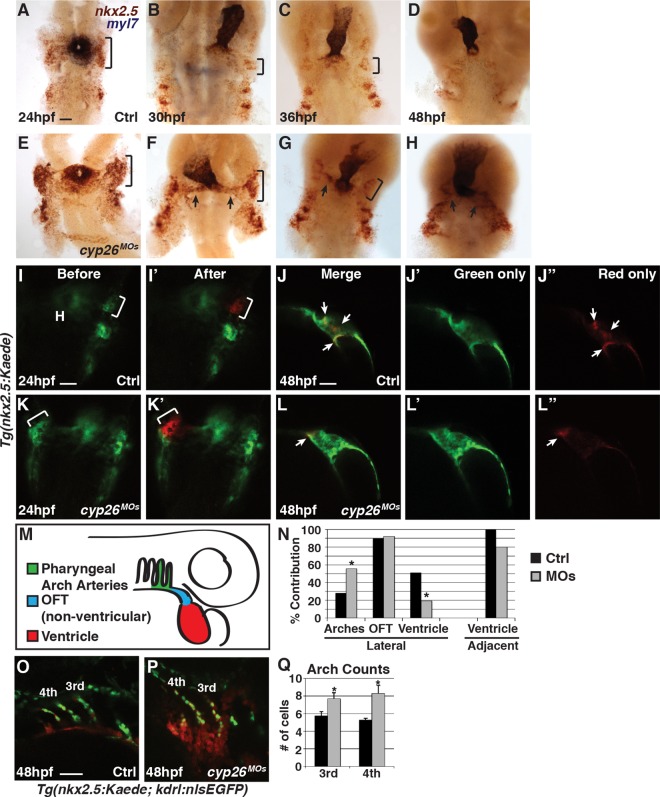
SHF progenitors do not add to the heart tube in Cyp26-deficient embryos. (A–H) ISH of control and Cyp26-deficient *Tg*(*nkx2*.*5*:*ZsYellow*) embryos at 24 (A,E), 30 (B,F), 36 (C,G), and 48 (D,H) hpf. *Nkx2*.*5*:*zsyellow* (red) and *myl7* (purple). (A–C,E–G) Brackets indicate *nkx2*.*5*:*ZsYellow+* cells in the first *nkx2*.*5+* pharyngeal arch. (F,G) Arrows indicate accumulation of *nkx2*.*5+* cells adjacent to the arterial pole of the heart. (I,I’,K,K’) *Tg(nkx2*.*5*:*Kaede)* before and after photoconversion of the anterior lateral population of *nkx2*.*5+* cells (bracket) in control and Cyp26-deficient embryos at 24 hpf. (J–J”,L–L”) Position of photoconverted cells (red, arrows) relative to the heart tube (green) in control and Cyp26-deficient embryos at 48 hpf. Images in I–J” and K–L”, respectively, are of the same control and Cyp26-deficient embryos. (M) Schematic depicting the three regions where photoconverted *nkx2*.*5*:*Kaede+* cells contributed at 48 hpf. (N) Graph depicting percentage of contribution to the third and fourth PAAs, non-ventricular OFT, and ventricular cardiomyocytes (control-lateral *n* = 39, control-adjacent *n* = 8, Cyp26-deficient–lateral *n* = 52, Cyp26-deficient–adjacent *n* = 5). (O,P) Confocal images of arch arteries in *Tg(nkx2*.*5*:*Kaede; kdrl*:*nlsEGFP)* control and Cyp26-deficient embryos. (Q) Graph depicting quantification of the endothelial cell number in the third and fourth PAAs in control (*n* = 18) and Cyp26-deficient (*n* = 24) embryos. Ventral view, anterior up (A–H); dorsal view, anterior up (I,I’,K,K’); lateral view, anterior up (J,J’,J”,L,L’,L”); lateral view, anterior right (O,P). *n* > 20 per group for (A–H). Asterisk denote *p* < 0.05 by Chi Squared test. H, heart. Scale bar: 50 μm.

To examine SHF progenitors at time points relevant to the observed SHF ventricular defects, we performed two-color ISH for *myl7* to mark the differentiated cardiomyocytes and *zsyellow* to track *nkx2*.*5+* undifferentiated SHF progenitors proximal to the heart tube using *Tg*(*nkx2*.*5*:*ZsYellow*) embryos. Cyp26-deficient embryos had an increase in *nkx2*.*5*:*zsyellow* expression lateral to the heart at stages from 24 through 36 hpf compared to control embryos (Fig 2A–2C and 2E–2G). After 36 hpf, we found that *nkx2*.*5*:*ZsYellow+* progenitors accumulated outside of the heart tube in Cyp26-deficient embryos (Fig 2G and 2H), suggesting a failure to add differentiated ventricular cardiomyocytes may be due to improper progenitor migration. To specifically test the ability of SHF progenitors to migrate and join the arterial pole of the heart, we used the *Tg(nkx2*.*5*:*Kaede)* line. Small clusters of *nkx2*.*5*:*Kaede+* cells located adjacent to the forming heart tube or more laterally were photoconverted at 24 hpf (Fig 2I, 2I’, 2K and 2K’). The location of the photoconverted red cells with respect to the heart tube was then recorded at 48 hpf (Fig 2J–2J”, 2L–2L”, 2M and 2N). *Nkx2*.*5*:*Kaede+* cells photoconverted adjacent to the nascent heart tube joined the heart tube in both control and Cyp26-deficient embryos at 48 hpf, although the frequency was reduced in Cyp26-deficient embryos (Fig 2N). However, photoconverted *nkx2*.*5*:*Kaede+* cells of the lateral populations, which have previously been shown to also give rise to the PAAs [35], ingressed into the heart significantly less frequently and more frequently contributed to the third and fourth PAAs of the branchia in Cyp26-deficient embryos (Fig 2I–2N). To determine if there is an increase in endothelial cells that contributed to these arch arteries, we crossed the *Tg(nkx2*.*5*:*Kaede)* and *Tg(kdrl*:*nlsEGFP)* lines to facilitate counting of the *nkx2*.*5+* endothelial cells via their nuclei. We found that Cyp26-deficient embryos had an increase in the number of endothelial cells in the third and fourth arch arteries (Fig 2O–2Q), suggesting that some SHF progenitors may differentiate as arch artery endothelial cells at the expense of becoming ventricular cells in Cyp26-deficient embryos. Therefore, SHF progenitors fail to migrate and add appropriately to the extending OFT of the heart tube and more frequently contribute to the anterior PAAs in Cyp26-deficient embryos.

### Differentiated Ventricular Cardiomyocytes Are Extruded from the Heart Tube in Cyp26-Deficient Embryos

**Fig 3:**
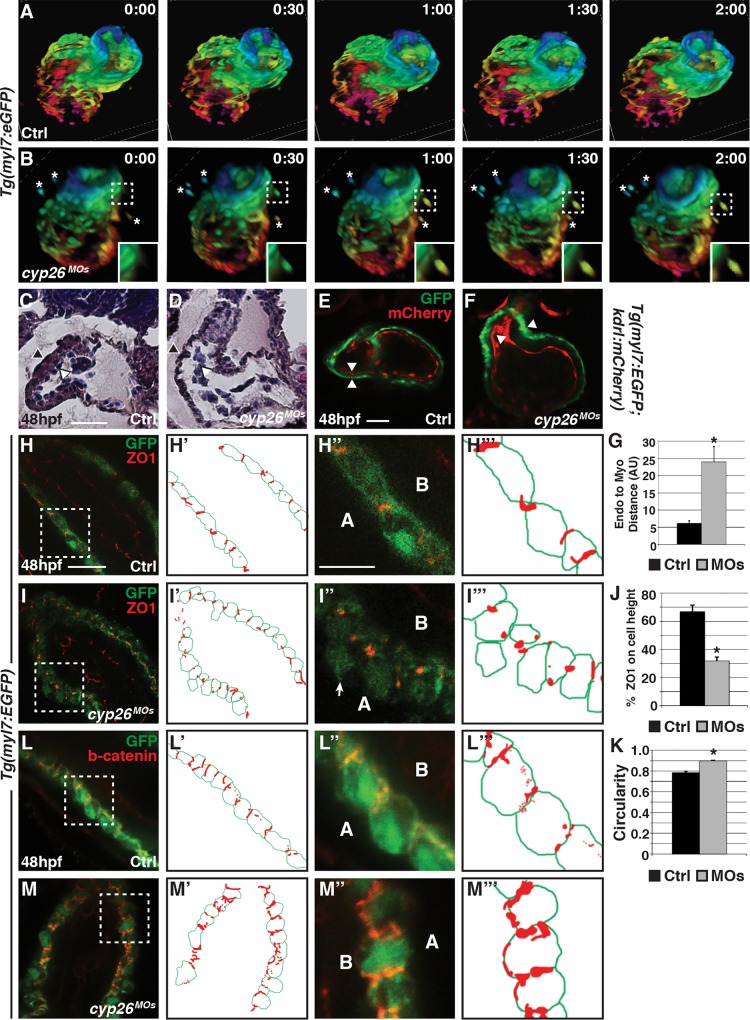
Ventricular cardiomyocytes can exit the heart tube in Cyp26-deficient embryos. (A,B) 30-min interval frames from confocal time-lapse movies of control and Cyp26-deficient *Tg(myl7*:*EGFP)* hearts. Images of hearts were depth-coded with the spectrum ranging from pink at 120 μm to blue at 0 μm. (C,D) Hematoxylin–eosin (HE) stained frontal sections of the hearts from control and Cyp26-deficient embryos. Endocardium (white arrowheads) and myocardium (black arrowheads). Control hearts *n* = 4. Cyp26-deficient hearts *n* = 5. (E,F) Control and Cyp26-deficient *Tg(myl7*:*EGFP);Tg*(*kdrl*:*mCherry*) embryos at 48 hpf. Endocardium (red) and myocardium (green). Arrowheads indicate the inner border of endocardial and outer border of myocardial cells. (G) Graph showing the distance between the endocardial and myocardial layers (control *n* = 5, Cyp26 deficient *n* = 7). (H–I”‘) Confocal images of control and Cyp26-deficient *Tg(myl7*:*EGFP)* hearts stained for zonula occludens 1 (ZO1) (red) and green fluorescent protein (GFP) (green), with schematized outlines of cell boundaries and ZO1 staining. Arrow denotes cardiomyocyte protruding into the pericardial space. (J) Graph depicting the percentage of ZO1 expression along the height of cardiomyocytes (control *n* = 15, Cyp26 deficient *n* = 15). (K) Graph depicting circularity measurement of ventricular cells (control *n* = 15, Cyp26 deficient *n* = 30). (L–M”‘) Confocal images of control and Cyp26-deficient *Tg(myl7*:*EGFP)* hearts stained for β-catenin (red) and GFP (green), with schematized outlines of cell boundaries and β-catenin staining. Error bars are SEM, asterisk denotes *p* < 0.05 by Student’s *t* test. Frontal views, anterior up (A–F,H,I,L,M); *n* > 20 embryos per group (E,F,H,I,L,M). A, apical; B, basal. Scale bar for C–M’: 50 μm. Scale bar for H”–M”‘: 25 μm.

Although a failure of SHF addition can partially account for the ventricular cardiomyocyte OFT defects in Cyp26-deficient embryos, the cardiomyocyte counts indicate that cells from the mainly FHF-derived heart tube may also be lost, as we observe fewer ventricular cardiomyocytes at 48 and 72 hpf compared to 36 hpf in Cyp26-deficient embryos (Fig 1G). Immunohistochemistry (IHC) for activated Caspase 3 (aCasp3) indicated there is not a significant increase in apoptosis within the heart tube of Cyp26-deficient embryos compared to control embryos (S6A and S6B Fig). Thus, cell death within the heart tube does not explain the loss of ventricular cardiomyocytes. However, quite surprisingly, we noticed cardiomyocytes outside the heart tube in ~50% (28/61) of Cyp26-deficient *Tg(myl7*:*EGFP)* embryos (S6B and S6B” Fig), as well as in *Tg(myl7*:*Kaede)* embryos (Fig 1J). In contrast, cardiomyocytes were never observed outside of the heart in control embryos. Furthermore, ectopic *myl7*:*EGFP+* cardiomyocytes were also observed in *gir* and *gir+c1* embryos (S2K, S2L and S2P Fig) as well as RA-treated embryos (S3E Fig). Importantly, during the *myl7*:*Kaede* assays to address cardiomyocyte addition, we found that the ectopic cardiomyocytes in Cyp26-deficient embryos fluoresced red (Fig 1J), indicating that these cardiomyocytes were differentiated prior to 36 hpf and likely predominantly derived from the FHF. To determine if the ectopic cardiomyocytes are exiting the heart tube, we performed time-lapse imaging from 40 hpf to 52 hpf with confocal microscopy using *Tg(myl7*:*EGFP)* embryos. This analysis revealed that ventricular cardiomyocytes can be extruded from the differentiated heart tube into the pericardial space in Cyp26-deficient embryos, a phenomenon never observed in control siblings (Fig 3A and 3B; S1 and S2 Movies). Although the aCasp3 IHC did not label cells within the heart tube, we found that the ectopic cardiomyocytes within the pericardial space were co-labeled for aCasp3 and *myl7*:*EGFP+* (S6A–S6B” Fig), suggesting cardiomyocytes that exit the heart undergo apoptosis. Therefore, the loss of ventricular cardiomyocytes in Cyp26-deficient embryos is, at least in part, due to differentiated FHF cardiomyocytes exiting the heart and dying.

### Ventricular Cardiomyocytes Have Disrupted Polarity in Cyp26 Deficient Embryos

To begin to understand the cellular mechanisms underlying the ectopic cardiomyocytes in Cyp26 deficient embryos, we examined sectioned hearts with hematoxylin–eosin (HE) staining and confocal sections of *Tg(myl7*:*EGFP; kdrl*:*mCherry*) hearts. At 48 hpf, both assays revealed an uneven ventricular myocardium with an increased separation between the endocardium and myocardium in Cyp26-deficient embryos (Fig 3C–3G), suggesting adhesion between the myocardial and endocardial cell layers is disrupted. To further investigate ventricular cardiomyocyte adhesion and polarity, we performed IHC with ZO1 and β-catenin in *Tg(myl7*:*EGFP)* embryos. In Cyp26-deficient and *gir+c1* embryos, the ventricular cardiomyocytes of the heart tube were more round (Fig 3K and S7F Fig) and separated from each other, with some cardiomyocytes extruded from the single cell layer in the pericardial space (Fig 3I and 3I” and S7D Fig). The ventricular cardiomyocytes also had punctate ZO1 expression (Fig 3H–3J and S7A–S7E Fig) and more broadly expressed, mislocalized β-catenin in Cyp26-deficient embryos compared to control siblings (Fig 3L–3M”‘). Altogether, these data indicate that disruption of myocardial cell polarity and shape may facilitate ventricular cardiomyocyte extrusion from the heart tube in Cyp26-deficient embryos.

### Restoring FGF Signaling Can Partially Rescue Ventricular Defects in Cyp26-Deficient Embryos

**Fig 4:**
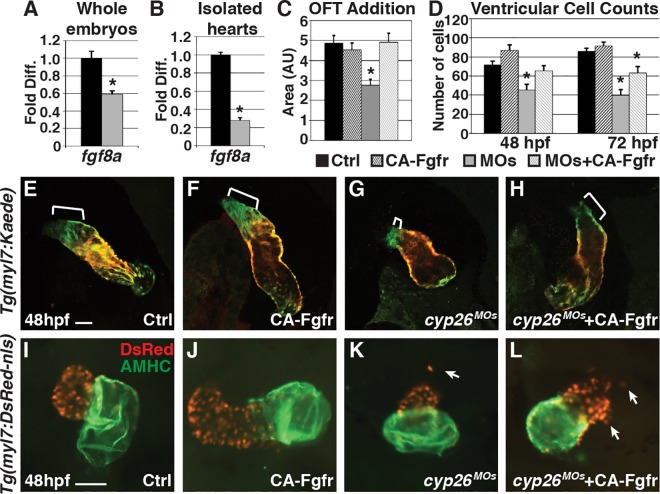
Restoring FGF signaling rescues SHF addition but not ventricular integrity. (A,B) Graphs indicating fold difference of mRNA relative to *β-actin* assayed with RT-qPCR of *fgf8a* expression in whole embryos and isolated hearts at 48 hpf. (C) Graph depicting quantification of ventricular addition to the OFT (control *n* = 8, CA-Fgfr *n* = 6, Cyp26 deficient *n* = 10, Cyp26 deficient+CA-Fgfr *n* = 6). (D) Graph of ventricular cardiomyocyte counts at 48 and 72 hpf (*n* = 10 per group). (E–H) Confocal images of optical slices from hearts of *Tg(myl7*:*Kaede)* and *Tg(myl7*:*Kaede);Tg(hsp70*:*ca-fgfr1)* embryos at 48 hpf after heat-shock at 24 hpf and photoconversion at 36 hpf. Brackets indicate added ventricular cells (green only). (I–L) IHC for hearts from *Tg*(*myl7*:*DsRed-NLS*) and *Tg*(*myl7*:*DsRed-NLS*);*Tg(hsp70*:*ca-fgfr1)* at 48 hpf after heat-shock at 24 hpf. Arrows indicate ectopic cardiomyocytes. Error bars are SEM, asterisk denotes *p* < 0.05 by Student’s *t* test. Frontal views with anterior up (E–L); *n* > 20 embryos per group (E–L). Scale bar: 50 μm.

Next, we sought to identify molecular mechanisms underlying the ventricular OFT defects in Cyp26-deficient embryos. We first examined *fibroblast growth factor 8a* (*fgf8a*), because previous studies have shown that RA and FGF exhibit a mutually antagonistic relationship in the heart [36,37], and FGF is required in vertebrates for SHF development through promoting proper proliferation and migration [38–41]. Indeed, *fgf8a* expression was significantly decreased in Cyp26-deficient embryos and their isolated hearts at 48 hpf (Fig 4A and 4B). Furthermore, within the lateral *nkx2*.*5+* cell population, which encompasses the SHF progenitors, proliferation is decreased, consistent with previous findings on loss of FGF signaling (S5M Fig) [38–40]. Restoring FGF signaling in Cyp26-deficient embryos at 24 hpf through heat-shock–induced expression of a constitutively active FGF receptor using the transgenic line *Tg(hsp70*:*ca-fgfr1)* can restore ventricular addition (Fig 4C and 4E–4H) and ventricular cardiomyocyte number at 48 hpf (Fig 4D and 4I–4L). Therefore, these data suggest that the loss of FGF signaling contributes to the ventricular OFT defects in Cyp26-deficient embryos.

Despite the ability of induced FGF signaling to rescue some aspects of OFT addition in Cyp26-deficient embryos, heart morphology and the appearance of ectopic cardiomyocytes was not significantly restored (Fig 4I–4L; S8A and S8B Fig). Furthermore, there was still a significant decrease in ventricular cardiomyocyte number (Fig 4D) and loss of *elnb* expression at 72 hpf in FGF-restored Cyp26-deficient embryos (S4E–S4H Fig). An additional induction of FGF signaling at 48 hpf also did not restore ventricular cardiomyocyte number (S8C Fig). Together, these results suggest that loss of FGF signaling can only partially explain ventricular OFT defects and that additional effectors must contribute to the OFT defects in Cyp26-deficient embryos.

### Excess Matrix Metalloproteinase Expression Impairs SHF Addition and OFT Integrity

**Fig 5:**
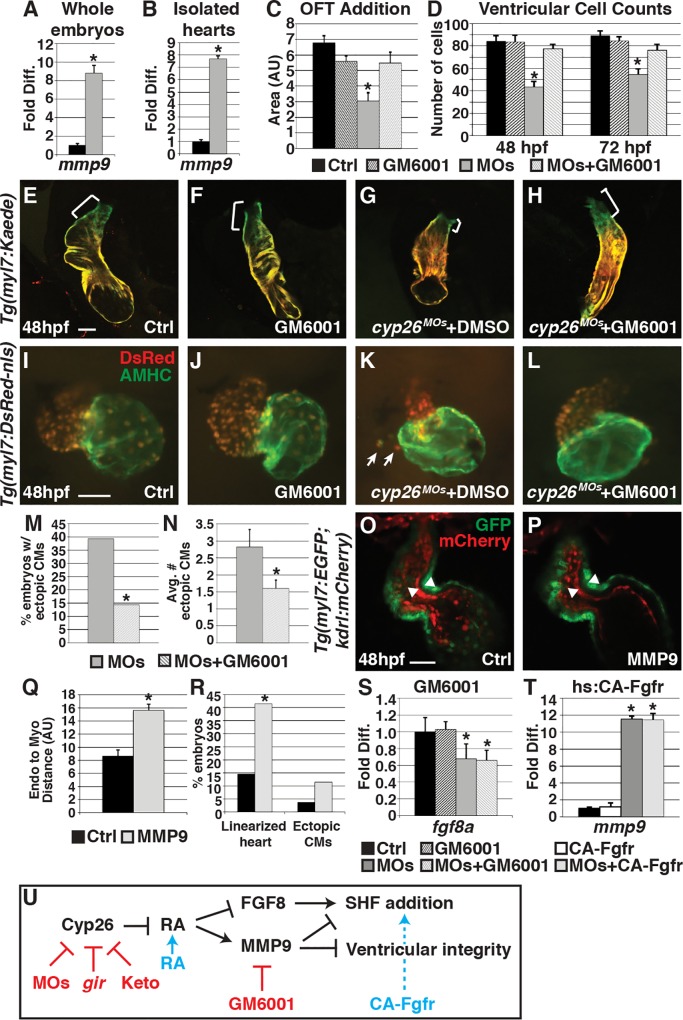
Attenuating MMP function restores SHF addition and ventricular integrity. (A,B) Graphs indicating fold difference of mRNA relative to *β-actin* assayed with RT-qPCR of *mmp9* expression in whole embryos and isolated hearts at 48 hpf. (C) Graph showing quantification of ventricular addition to the OFT (control *n* = 11, GM6001 treated *n* = 11, Cyp26 deficient *n* = 12, Cyp26 deficient + GM6001 *n* = 12). (D) Graph depicting ventricular cardiomyocyte counts at 48 and 72 hpf (*n* = 10 per group). (E–H) Confocal images of optical slices from hearts of *Tg(myl7*:*Kaede)* embryos at 48 hpf after photoconversion at 36 hpf. Brackets indicate ventricular addition (green only). (I–L) IHC of hearts from control and Cyp26-deficient *Tg*(*myl7*:*DsRed-NLS*) embryos after DMSO or GM6001 treatment. Arrows indicate ectopic cardiomyocytes. (M) Graph depicting the percentage of embryos with ectopic cardiomyocytes in Cyp26 deficient treated with DMSO or GM6001 at 48 hpf. (N) Graph depicting average number of ectopic cardiomyocytes (per embryo with ectopic cardiomyocytes) at 48 hpf. (O,P) Confocal images of optical slices through control (lineage tracer alone) or activated MMP9-injected *Tg(myl7*:*EGFP);Tg*(*kdrl*:*mCherry*) hearts at 48 hpf. Arrowheads denote the inner border of endocardial and outer border of myocardial cells. (Q) Graph depicting the quantification of the distance between the endocardium and myocardium (control *n* = 12, MMP9 injected *n* = 10). (R) Graph depicting the percentage of embryos with linearized, dysmoprhic hearts and cells outside the heart (*n* > 50 per group). (S) Graph indicating fold difference of mRNA relative to *β-actin* assayed with RT-qPCR of *fgf8a* expression at 48 hpf in control and Cyp26-deficient embryos treated with DMSO or GM6001. (T) Graph indicating fold difference of mRNA relative to *β-actin* assayed with RT-qPCR of *mmp9* expression at 48 hpf after heat-shock induction of CA-Fgfr1. (U) Model of Cyp26 enzyme function in the ventricular OFT development. Red indicates reagents used to inhibit function. Blue indicates reagents used to activate function. Controls in C–E, I, and S were DMSO treated. Controls in O, Q, R indicate Cascade blue-dextran injected alone. Error bars are SEM, asterisks denote *p* < 0.05 compared to controls by Student’s *t* test (A–D,N,Q–T), asterisk denotes p<0.05 by Chi Squared test (M). Frontal views, anterior up (E–L,M,N); *n* > 20 embryos per group (E–L,M,N). CMs, cardiomyocytes. Scale bar: 50 μm.

Because a loss of *fgf8a* expression cannot completely account for the OFT defects in Cyp26-deficient embryos, we next wanted to identify candidate effectors downstream of RA signaling that could explain the ventricular cardiomyocyte polarity and heart tube integrity defects. We examined *matrix metalloproteinase 9* (*mmp9*) expression, a gelatinase that breaks down collagen in the extracellular matrix [42], because previous studies have shown that RA signaling can induce its expression in several disease contexts [43–46]. Furthermore, reminiscent of our observations of ventricular cells in Cyp26-deficient embryos, excess MMP9 disrupts the structural integrity of the vasculature [47] and lung epithelium [48]. Using RT-qPCR and ISH, we found MMP9 expression was significantly increased in Cyp26-deficient whole embryos and their isolated hearts (Fig 5A and 5B; S9A and S9B Fig). *Gir* mutants and *gir+c1* embryos also had increased *mmp9* expression (S9C Fig). Furthermore, the increased *mmp9* expression in Cyp26-deficient embryos is not due to an increase in macrophages or neutrophils (S9D–S9I Fig), which are known to secrete MMP9 [49].

To determine if attenuating MMP function can rescue SHF addition, embryos were treated with a 25-μM concentration of the MMP inhibitor GM6001 [50] beginning at 6 hpf and assayed for ventricular cardiomyocyte addition and number. We found that attenuating MMP function restored SHF addition (Fig 5C and 5E–5H) and ventricular cardiomyocyte number at 48 hpf (Fig 5D). In contrast to induction of FGF signaling, heart morphology was markedly improved (Fig 5I–5L), ectopic cardiomyocytes were less frequently observed (Fig 5M and 5N), and ventricular cardiomyocyte polarity and shape were restored (S10A–S10F Fig) in GM6001-treated Cyp26-deficient embryos compared to DMSO-treated siblings. Moreover, ventricular cardiomyocyte number was better maintained (Fig 5D), and bulbous arteriosus formation was restored in GM6001-treated Cyp26-deficient embryos through 72 hpf (S4I–S4L Fig). To further test the sufficiency of increased MMP to cause OFT defects, we injected activated human MMP9 protein into the pericardial space of 24 hpf *Tg(myl7*:*EGFP*; *kdrl*:*mCherry*) embryos. A small but significant percentage of MMP9-injected embryos had heart defects similar to Cyp26-deficient embryos that included more linear heart morphology, separation of the endocardium from the myocardium, and ectopic cardiomyocytes (Fig 5O–5R). Altogether, these data suggest that increased MMP activity in Cyp26-deficient embryos likely disrupts the extracellular environment, which impairs SHF addition and ventricular integrity in Cyp26-deficient embryos.

### FGF and MMP Work in Parallel Downstream of Excess RA Signaling

Finally, we wanted to determine the hierarchical relationship of these perturbed molecular signals downstream of RA signaling. In Cyp26-deficient embryos, excess MMP9 appeared to underlie both the RA-induced ventricular OFT addition and integrity loss, whereas loss of Fgf8a was only partially responsible for the ventricular addition defects. Thus, because excess MMP9 can account for more of the RA-induced OFT defects than loss of Fgf signaling, we postulated that increased MMP9 may be upstream and contribute to the loss of *fgf8a* expression in Cyp26-deficient embryos. In contrast to this hypothesis, attenuating MMP function did not restore *fgf8a* expression in Cyp26-deficient embryos (Fig 5S), and, conversely, restoring FGF signaling did not affect *mmp9* expression (Fig 5T). Therefore, our data support a model in which *fgf8a* and *mmp9* act in parallel downstream of RA signaling in Cyp26-deficient embryos (Fig 5U).

## Discussion

Our studies have provided insight into previously unappreciated cellular and molecular mechanisms of RA-induced OFT defects. We find that excess embryonic RA levels from endogenous sources due to Cyp26 deficiency inhibit ventricular OFT development after initial heart tube formation. The loss of proper ventricular OFT development in Cyp26-deficient embryos is due to both a failure of ventricular SHF progenitor addition and loss of differentiated FHF-derived ventricular cells from the initial heart tube. Furthermore, SHF progenitors more frequently contribute to and differentiate as third and fourth PAA cells in Cyp26-deficient embryos, which has not been described previously. Although loss of FGF signaling plays a role downstream of excess RA in the generation of OFT defects, we identified that excess MMP9 is a primary effector of ventricular OFT defects in Cyp26-deficient embryos. Thus, our studies support the hypothesis that disruption of the extracellular environment is a key cause of RA-induced ventricular OFT defects after the initial stages of heart development.

In many developmental contexts, RA signaling has an antagonistic relationship with FGF signaling. During patterning of the cardiac progenitor field, previous studies have shown that RA signaling establishes the posterior boundary of the SHF in mice and zebrafish [37,51,52]. At slightly later stages, FGF signaling is required to promote SHF progenitor proliferation and deployment [39,40,53]. Our studies indicate that it is critical to limit RA signaling after the initial patterning of FHF progenitors to promote FGF signaling that directs SHF progenitor development, providing another developmental context for the antagonistic relationship between these essential pathways. Consistent with previous analysis of FGF signaling in SHF development [39,53], we found that there is decreased proliferation in SHF progenitors. However, in comparing the ventricular OFT defects in Cyp26- and FGF signaling–deficient embryos, it is noteworthy that the loss of ventricular cardiomyocytes in Cyp26-deficient embryos is much greater than the loss of ventricular cardiomyocytes previously reported for FGF signaling deficient embryos (>50% loss in Cyp26-deficient versus ~20% loss in FGF signaling–deficient embryos) [54]. Importantly, restoration of FGF signaling in Cyp26-deficient embryos only partially restored ventricular cardiomyocyte number and did not restore ventricular integrity. Thus, our interpretation is that decreased FGF signaling in SHF progenitors is only one mechanism that contributes to RA-induced OFT defects in vertebrates.

A key finding of our study is that perturbation of the extracellular environment, which we posit in turn adversely affects ventricular cardiomyocyte polarity, is one of the main factors contributing to OFT defects from surplus RA signaling. Previous studies of fibronectin levels in zebrafish have shown that a proper extracellular matrix environment is required for FHF progenitor migration to the midline during somitogenesis [55,56]. In mice, fibronectin is required for SHF development, with loss of fibronectin resulting in shortened OFTs [57]. Interestingly, previous studies also found that excess RA signaling can disrupt the extracellular matrix in the endocardial cushions, leading to transposition of the great arch arteries [58,59]. However, the mechanisms underlying extracellular matrix disruption in this context are not understood. Our data implicate a necessity to limit MMP9 levels within the heart tube and surrounding environment. Although our study is the first to our knowledge to designate misregulation of MMP9 as a crucial effector of RA-induced OFT defects, exogenous RA signaling promotes MMP9 expression in several other developmental and disease contexts, including dendritic cell migration [60], neuroblastoma [43,44], and glomerulosclerosis [45]. Although there are multiple conserved RA response elements in the promoter of MMP9 [61], future experiments will be necessary to determine if RA signaling directly promotes MMP9 expression within the pharyngeal tissues and heart. Interestingly, recent studies of Ezh2, a component of the Polycomb repressive complex 2 (PRC2), have indicated a necessity to limit MMP9 expression in vascular development, as excess MMP9 can result in loss of vascular integrity [47]. Tie2-mediated conditional knockdown of Ezh2 in endothelial cells results in a separation of the endocardium from the myocardium observed at E11.0 [47], similar to what we observe in Cyp26-deficient embryos. Additionally, direct application of MMP9 to the lung epithelium causes loss of tight junctions and cell extrusion [48], which is highly reminiscent of the ventricular cardiomyocyte polarity and integrity defects we observe in Cyp26-deficient embryos. Although we do not find a direct transcriptional relationship between *fgf8a* and *mmp9*, it is interesting that a recent study found that Cadm4, which is involved in cell adhesion, is a downstream effector of FGF signaling in OFT development [38], suggesting FGF signaling may promote critical interactions with the extracellular environment. Future experiments will determine if RA-induced MMP9 expression directly perturbs signals known to promote proper SHF development, including FGF, Tgf-β ligands and their receptors, and cell adhesion molecules.

RA signaling has been shown to function in a regulatory loop with Tbx1 and FGF signaling. Disruption of this regulatory network is thought to underlie malformations in DiGeorge Syndrome, at least in the posterior pharyngeal mesoderm [62]. Additionally, cell polarity and tight junction defects have recently been described in SHF progenitors of Tbx1 null mice [63]. Although previous studies have found RA signaling negatively regulates *tbx1* expression during early somitogenesis [64,65], via RT-qPCR and ISH, we found that *tbx1* expression is not reduced or significantly altered in Cyp26-deficient embryos at these later stages (S5N–S5R Fig). Therefore, we have identified unanticipated temporal sensitivity to the canonical Tbx1-RA signaling regulatory loop [15,65–67], suggesting that transcriptional repression of *tbx1* due to excess RA signaling cannot explain the later ventricular cardiomyocyte OFT defects in Cyp26-deficient embryos presented here. Moreover, previous genetic interactions between RA signaling and Tbx1 have been shown to affect the most posterior pharyngeal arteries through affecting the neural crest in mice [67–71]. Thus, our data are the first to indicate that excess RA signaling can promote a surplus of endothelial cells in the more anterior arch arteries, potentially at the expense of ventricular SHF progenitors and independent of the canonical Tbx1-RA regulatory mechanism.

Altogether, our study provides critical insight into the sensitivity of OFT development to excess embryonic RA levels, which can occur from genetic loss of Cyp26 enzymes or exogenous RA treatment. Furthermore, we found that excess RA signaling at later stages of development than previously appreciated can disrupt ventricular cells derived from both the FHF and SHF. Importantly, our study is also the first, to our knowledge, to implicate excess MMP9 and consequently disruption of the extracellular environment as primary effectors of RA-induced CHDs. The function of Cyp26 enzymes and the necessity to limit RA signaling is a highly conserved aspect of vertebrate heart development. Therefore, our findings significantly enhance understanding of the molecular and cellular etiologies of developmental syndromes with RA-induced OFT defects.

## Materials and Methods

### Ethics Statement

All zebrafish husbandry and experiments were performed under conditions outlined in protocols approved by IACUC and Cincinnati Children’s Hospital Medical Center.

### Zebrafish Husbandry and Strains

Adult zebrafish were grown and maintained under standard laboratory conditions [72]. Transgenic lines used were: *Tg(β-actin*:*VPBD-RLBD;UAS*:*EGFP)* [32], *Tg*(*–5*.*1myl7*:*DsRed2-NLS*)^*f2*^ [73], *Tg*(*−6*.*5kdrl*:*mCherry*)^*ci5*^ [74], *Tg(myl7*:*Kaede)*^*sd22*^ [33], *TgBAC*(*−36nkx2*.*5*:*ZsYellow*)^*fb7*^ [7], *Tg(nkx2*.*5*:*Kaede)*^*fb9*^ [75], *Tg(kdrl*:*nlsEGFP)* [76], *Tg(myl7*:*EGFP)*^*twu*^ [77], and *Tg(hsp70*:*ca-fgfr1)*^*pd3*^ [54]. The *giraffe/cyp26a1* mutant line [25] was used.

### MO and mRNA Injections

MO injections were performed at the one-cell stage as described previously [16]. MO sequences for *cyp26a1* and *cyp26c1* were previously published [24,78]. Knockdown of *cyp26a1* and *cyp26c1* together was attained using a cocktail of 1.5 ng *cyp26a1* MO1, 0.75 ng *cyp26a1* MO2, and 4 ng *cyp26c1*. For experiments using the *gir* mutant embryos, heterozygous carriers were in-crossed, and the resulting embryos were either injected using 4 ng *cyp26c1* MO or kept as uninjected controls. Nonspecific MO-induced cell death was counteracted with 2 ng *p53* MO for all injections. The *cyp26a1* mRNA was injected at 500 pg as previously described [79,80].

### IHC and Cardiomyocyte Counts

IHC and cardiomyocyte counts were performed using *Tg*(*myl7*:*DsRed-NLS*) embryos as described previously [22]. For cell counts and cell polarity markers, embryos were fixed with 1% Formaldehyde in 1X PBS for 1 h then washed with 0.2% Saponin in 1X PBS for 3X at 5 min. Embryos were blocked with Saponin Blocking solution (1X PBS, 10% sheep serum, 2 mg/ml BSA and 0.2% Saponin) for 1 h then incubated with primary antibody at 4°C overnight. Embryos were washed with 0.2% Saponin in PBS 3X at 5 min then incubated with secondary antibodies in Saponin Blocking solution for 2 h at room temperature. Embryos were washed with 0.2% Saponin in 1X PBS 3X at 5 min then imaged or stored at 4°C for up to 1 wk. Primary antibodies used were anti-DsRed2 1:1000 (632496, Clontech), anti-sarcomeric myosin (MHC)/MF20 1:10 (gift of D. Yelon), anti-atrial myosin heavy chain (AMHC)/S46 1:10 (University of Iowa Hybridoma Bank), anti-GFP chick 1:250 (ab13970, Abcam), anti-ZO1/TJP11:250 (33–9100, Life Technologies), and anti-β-catenin 1:100 (C7207-100UL, Sigma). Secondary antibodies for goat anti-chicken IgG-FITC (6100–02, Southern Biotech), Goat anti-mouse IgG1 TRITC (1070–02, Southern Biotech), Goat anti-mouse IgG1 FITC (1070–02, Southern Biotech), goat anti-rabbit IgG-TRITC (4050–02, Southern Biotech) and Goat anti-mouse IgG2b TRITC (1090–03, Southern Biotech) were all used at 1:100.

For detection of aCasp3 and phospho-Histone H3 (pHH3) staining, embryos were fixed in 4% paraformaldehyde at 4°C overnight then dehydrated with a methanol series to 100% methanol and left in the -20°C freezer for at least 2 h. Embryos were rehydrated using PBST (PBS with 1% tween) then permeabilized using PDT (PBST with 1% DMSO) supplemented with 0.3% Triton-X for 20 min at room temperature. Embryos were then blocked with 1xPBS/0.1%Tween20/10% sheep serum (013-000-121 Jackson Immunoresearch) for 30 min followed by incubation in primary antibodies overnight at 4°C. Embryos were rinsed 3X for 20 min with PDT then reblocked for 30 min and incubated in secondary antibodies for 2 h at room temperature. Embryos were washed 3X for 5 min with PDT then imaged or stored at 4°C for up to 1 mo. Primary antibodies used were rabbit anti-active Caspase-3 1:250 (559565, BD Biosciences Pharmigen) and anti-histone H3 1:750 (ab14955, Abcam). Secondary antibody used was goat anti-rabbit IgG-TRITC (4050–02, Southern Biotech) at 1:100 dilution. For both methods, embryos were imaged using either a Zeiss M2BioV12 fluorescent stereomicroscope or Nikon A1 confocal microscope.

To count *nkx2*.*5+* cells in the cardio-pharyngeal region and pHH3+ cells, the IHC was performed as described above. Embryos were oriented dorsal side down in a small well created by using a capillary tube to punch a hole in a layer of 2% agarose within a μ-Slide 2-well (Ibidi) followed by imaging with a Nikon A1 confocal microscope. Images were analyzed using Imaris software (Bitplane). Despite the absence of a nuclear tag *nkx2*.*5*:*ZsYellow* the individual nuclei of cells were still clearly visible, which facilitated the creating of spots in Imaris and cell counting.

### Drug Treatments

Stocks of 10 mM Ketoconazole (BP2734-50, Fisher), 100 mM RA, and 1 mM DEAB (4-diethylaminobenzaldehyde; Sigma) were dissolved in DMSO (Sigma). Embryos were treated at a final concentration of 25 μM Ketoconazole in embryo water starting at two-cell stage. Embryos were treated with a final concentration of 2 μM RA in embryo water starting at 24 hpf. Embryos were treated with a final concentration of 25 nM DEAB in embryo water starting at 50% epiboly. GM6001 (CC1010, Millipore) was purchased in a 2.5 mM stock in DMSO. Embryos were treated with 25 μM GM6001 in embryo water starting at 50% epiboly.

### Real-Time Quantitative PCR (RT-qPCR)

cDNA preparation and RT-qPCR was performed as previously described [16,78]. Briefly, embryos were lysed in Trizol then RNA was isolated using the PureLink RNA Micro Kit (Invitrogen). cDNA was obtained using the Thermoscript RT-PCR System (Invitrogen). RT-qPCR was performed under standard conditions using SYBR green PCR master mix (Applied Biosystems) in a Bio-Rad CFX PCR machine. Expression levels of *myl7*, *vmhc*, *mef2cb*, *ltbp3*, *nkx2*.*5*, *fgf8a*, and *mmp9* were standardized to *β-actin*. Data were analyzed using 2^−ΔΔCT^ Livak Method and Student's *t* test. Primer sequences for *myl7*, *vmhc*, *nkx2*.*5*, and *β-actin* were previously reported [16]. Primer sequences for *ltbp3* were previously reported [7]. Primer sequences for *fgf8a*, *mef2cb*, *mmp9*, and *tbx1* are as follows: *fgf8a* F-aatccggacctaccagcttt and R-atcagtttccccctcctgtt, *mef2cb* F-ctatggaaaccaccgcaact and R-tgcgcagactgagagttgtt, *mmp9* F-caaatctgtgttcgtgacgttt and R-tccgtcgaatgtcttgtagttg, and *tbx1* F-tattccggatccaactcagc and R-taatctgccattgggtccat.

### ISH

Whole mount ISH was performed as previously described [81]. Probes for *mef2cb* (ZDB-GENE-040901-7), *ltbp3* (ZDB-GENE-060526-130), *zsyellow* (accession number: Q9U6Y4), *nkx2*.*5 (*ZDB-GENE-980526), *myl7* (formerly *cmlc2*; ZDB-GENE-991019), *mfap4* (ZDB-GENE-040426-2246), and *mpx* (ZDB-GENE-030131-9460) were previously reported. The *elnb* probe was cloned from cDNA using primers F′-cagaggcaaaagctgcaaaatatg and R′-atccttgaccaaatcctccagcgg and placed into the pGemT-easy vector (Promega). The *mmp9* probe was cloned from cDNA using primers F′-tgacgggaacagcaatgaagc and R′-tggagaaggtttcgttggcac and placed into pGemT-easy vector (Promega). Probe was synthesized using standard methods. Two-color ISHs were performed as described previously [82] using INT-BCIP (Roche). Embryos were de-yolked, flatmounted, and imaged using a Zeis M2BioV12 stereomicroscope.

### Histology

Fixed embryos were embedded in paraffin and sectioned at 7-μ thickness. HE was performed using the HE stain kit (American Master Tech) as directed by kit protocol.

### Heart Isolations

Heart isolations were performed as described previously [83].

### Heat-Shock Experiments

Heat-shock experiments were performed using hemizygous *Tg(hsp70*:*ca-fgfr1)* carriers crossed to either *Tg(myl7*:*Kaede) or Tg*(*–5*.*1myl7*:*DsRed-NLS*) adult fish. Resulting embryos were raised at 28.5°C until 24 hpf then they were heat-shocked at 37°C for 30 min in a BioRad Thermal Cycler. Carriers of the *Tg(hsp70*:*ca-fgfr1)* transgene were identified by crystalline-RFP in the lens and sorted from non-carriers at 48 hpf using Nikon A1 confocal or at 72 hpf using a Zeiss M2BioV12 fluorescent stereomicroscope. Embryos were then used for either Kaede-addition experiments or cell counts.

### Kaede Photoconversion Assays

Photoconversion of Kaede from *Tg(myl7*:*Kaede)* embryos was achieved by exposing the embryos to fluorescent light from a DAPI filter on a Zeiss M2BioV12 fluorescent stereomicroscope until all green fluorescence was gone (approximately 15 min). At 48 hpf, embryos were anesthetized with Tricaine, gently compressed under a coverslip, and imaged using a Nikon A1 confocal microscope. Cardiomyocyte addition to the heart was analyzed using ImageJ to measure the area of green-only OFT.

Photoconversion of Kaede in specific groups of cells in *Tg(nkx2*.*5*:*Kaede)* embryos was performed by orienting embryos dorsal side up in a small well created by using a capillary tube to punch a hole in a layer of 2% agarose within a μ-Slide 2-well (Ibidi). Kaede+ cells from embryos were first imaged using the 20X objective on a Nikon A1 confocal microscope. Then, a specific cluster of Kaede+ cells was photoconverted using a 10X zoom of the 20X objective and the DAPI filter. Conversion of Kaede+ cells took approximately 30 s. The Kaede+ green and red cells from the embryos were then imaged again. Imaged embryos were maintained at 28.5°C until 48 hpf when they were scored for contribution to cardiomyocytes and imaged using a Nikon A1 confocal microscope.

### Endothelial Cell Counts

Counting of endothelial cells in the third and fourth PAAs was performed in hemizygous *Tg(nkx2*.*5*:*Kaede; kdrl*:*nlsEGFP)* embryos. Kaede was photoconverted to red, as described above, in *nkx2*.*5+* cells just prior to imaging. Embryos were then mounted laterally on a footed coverslip, and confocal images were taken at 1.5-μm intervals to generate 10–25 μm Z-stacks of the arches using a Nikon A1 confocal microscope. Reconstructions of the pharyngeal arches were created using NIS Elements. EGFP-expressing endothelial cells in the third and fourth PAAs were then manually counted.

### Time-Lapse Movies

Images for time-lapse movies were taken with a Nikon A1 confocal microscope. Embryos were mounted in a μ-Slide 2-well (Ibidi) with 0.6% low melt agar and oriented to provide a frontal view of the heart. Using NIS Elements software, a time-lapse program was set up where the Z-stacks were taken spanning 150 μm at 3 μm per step. Hearts were imaged every 15 or 30 min. After imaging, the Z-stacks were reconstructed into hearts, cropped, and depth coded using NIS Elements software.

### MMP9 Injections

*Tg(myl7*:*EGFP*; *kdrl*:*mCherry*) embryos were raised to 24 hpf then mounted laterally in 0.6% low melt agar. Embryos were injected into the pericardial space with either Cascade blue-dextran tracer (D-1976, Invitrogen) alone or along with 12 pg activated MMP9 protein (ab168863, Abcam). Embryos were left to recover for 15 min then removed from the low melt agar and transferred to fresh embryo water. Embryos were analyzed, fixed with 4% Formaldehyde in PBS, and imaged using a Nikon A1 confocal microscope at 48 hpf.

### Statistics and General Methods

All RT-qPCR was analyzed using 2^−ΔΔCT^ Livak Method and Student's *t* test. Circularity measurements were performed by measuring area and perimeter in ImageJ then calculating circularity as 4*piArea*/*perimeter*^2 and analyzed using Student's *t* test. Cell counts were analyzed using Student's *t* test. Contribution of *nkx2*.*5*:*Kaede* cells to the heart and arches was analyzed using a Chi Squared test.

## Supporting Information

S1 Fig Cyp26-deficient embryos have increased RA signaling. (A-B') ISH for *gfp* in WT and Cyp26-deficient *Tg(β-actin*:*VPBD-RLBD;UAS*:*EGFP)* embryos. Arrows in A’ and B’ indicate expression in the heart. (C-F) ISH for *cyp26a1* in WT and Cyp26-deficient embryos at 32 and 48 hpf. Arrows in C’ and C’ indicate increased expression in the cardiac region. (A,B,C,D,E,F) Lateral view, anterior left, (A’,B’,C’,D’) Dorsal view, anterior up; n&gt;20 embryos (A-F). Scale bars: 50 mm.
(TIF)

S2 Fig *Gir* mutants and *gir+c1* embryos fail to add ventricular cardiomyocytes and have ectopic cardiomyocytes. (A-H) IHC of hearts from control, Cyp26c1-depleted, *gir* mutant, and *gir+c1 Tg(myl7*:*DsRed-NLS)* embryos at 48 and 72 hpf. (I-P) Hearts from control, Cyp26c1-depleted, *gir* mutant, and *gir+c1 Tg(myl7*:*EGFP)* embryos at 48 and 72 hpf. Arrows denote ectopic cardiomyocytes. Ectopic cardiomyocytes were observed in 0/74 control, 1/80 Cyp26c1-depleted, 8/29 *gir*, and 7/31 *gir+c1* at 48 hpf. (Q) Graph depicting ventricular cardiomyocyte counts at 48 and 72 hpf (n = 10 per group). (R) Graph depicting atrial cardiomyocyte counts at 48 hpf (n = 10 per group). (S) Graph depicting atrial cardiomyocyte counts at 48 hpf (n = 10 per group) in control and Cyp26-deficient embryos. (T) Graph of ventricular addition to the OFT (n = 10 per group). (U-X) Control, *cyp26c1* MO injected, *gir*, and *gir+c1 Tg(myl7*:*Kaede)* embryo hearts at 48 hpf after photoconversion at 36 hpf. Brackets indicate ventricular addition (green only). Error bars are SEM, asterisks denote p&lt;0.05 compared to controls by Student’s t-test. All images are frontal views with anterior up; n&gt;20 embryos for (U-X). Scale bars: 50 mm.
(TIF)

S3 Fig Pharmacologically enhancing RA levels can recapitulate Cyp26-deficient heart defects, while inhibiting RA production and co-injecting *cyp26a1* mRNA can restore Cyp26-deficient heart defects. (A,B) IHC of DMSO or Ketoconazole (Keto) treated embryos at 48 hpf with ventricle in red (MHC) and atrium in green (AMHC). (C) Graph depicting ventricular counts of DMSO or Ketoconazole treated embryos at 48 hpf. (D,E) Hearts of *Tg(myl7*:*EGFP)* embryos at 72 hpf that were treated with DMSO or 2 μM RA beginning at 24 hpf. Arrows denote ectopic cardiomyocytes. (F) Graph of ventricular counts embryos at 48 and 72 hpf (n = 10 per group) of DMSO or 2 μM RA treatment beginning at 24 hpf. (G-J) Hearts at 48 hpf in control or Cyp26-deficient *Tg(myl7*:*EGFP)* embryos treated with DMSO or DEAB. (K) Graph depicting ventricular cell counts at 48 hpf for DMSO, DEAB, Cyp26-deficient + DMSO, and Cyp26-deficient + DEAB treated embryos. (L) Graph depicting ventricular cell counts at 48 hpf in control and Cyp26-deficient *Tg(myl7*:*DsRed-NLS)* embryos injected with *cyp26a1* mRNA. (M-P) Hearts at 48 hpf from control, *cyp26a1* mRNA-injected, Cyp26-deficient and Cyp26-deficient injected with *cyp26a1* mRNA *Tg(myl7*:*EGFP)* embryos. Error bars are SEM, asterisks denote p&lt;0.05 by Student’s t-test. All images are frontal views with anterior up; n&gt;20 embryos for (A,B,D,E,G-J,M-P). Scale bars: 50 mm.
(TIF)

S4 Fig SHF-derived bulbous arteriosus does not form in Cyp26-deficient embryos. (A-D) ISH for *elnb* in control, Cyp26c1-depleted, *gir* mutant, and *gir+c1* embryos. (E-H) ISH for *elnb* in control and Cyp26-deficient embryos after heat-shock induction of FGF. (I-L) ISH for *elnb* in control or Cyp26-deficient embryos treated with DMSO or GM6001.Control embryo in I were treated with DMSO. Lateral view, anterior up (A-L). Scale bar: 50 mm.
(TIF)

S5 Fig SHF progenitors are specified in Cyp26-deficient embryos. (A-F) ISH of *mef2cb* at 16 somites (s), 24, and 30 hpf in control and Cyp26-deficient embryos. (G,H) ISH of *ltbp3* at 30 hpf in control and Cyp26-deficient embryos. Arrows in C-H indicate expression in the OFT. (I) RT-qPCR for *mef2cb* at 16 s, 24, 36 and 48 hpf. (J) RT-qPCR for *ltbp3* at 16 s, 24, 36 and 48 hpf. (K) RT-qPCR for *nkx2*.*5* at 16 s, 24, 36 and 48 hpf. (L) Graph depicting total number of *nkx2*.*5+* cells at 24 hpf (n = 11 for control, n = 14 for Cyp26-deficient). (M) Graph depicting the percentage of proliferating *nkx2*.*5+* cells at 24 hpf (n = 11 for control, n = 14 for Cyp26-deficient). (N) RT-qPCR for *tbx1* at 16 s, 24, 36, and 48 hpf. For RT-qPCR, fold difference of mRNA was calculated relative to *β-actin*. (O-R) ISH for *tbx1* at 24 and 30 hpf in control and Cyp26-deficient embryos. Arrows indicate *tbx1* staining adjacent to the heart. Error bars are SEM, asterisks denote p&lt;0.05 by Student’s t-test. Dorsal view, anterior up (A-D), lateral view anterior right (E-H); n&gt;20 embryos per group for (A-H). Scale bars: 50 mm.
(TIF)

S6 Fig Ectopic cardiomyocytes in Cyp26-deficient embryos undergo apoptosis. (A,B) IHC of control or Cyp26-deficient *Tg(myl7*:*EGFP)* embryos for aCasp3 (red) and GFP (green). Arrows denote ectopic cardiomyocytes co-expressing aCasp3. 16/18 ectopic cardiomyocytes were co-labeled. Asterisk denotes aCasp3+ cell potentially within the heart tube. Frontal view, anterior up; Control n = 30. Cyp26-deficient n = 11 embryos. Scale bar: 50 mm.
(TIF)

S7 Fig *Gir* mutants and *gir+c1* embryos have disrupted polarity and cell shape. (A-D) Confocal images of IHC of control, Cyp26c1-depleted, *gir* mutant, and *gir+c1 Tg(myl7*:*EGFP)* stained for ZO1 (red), and GFP (green). (A’-D’) Schematized outlines of cell boundaries and ZO1 staining for images in A-D. Arrows denotes cardiomyocytes protruding into the pericardial space. (E) Graph depicting the percentage of ZO1 expression along the height of cardiomyocytes (n = 36 for each group). (F) Graph depicting circularity measurement of ventricular cells (n = 36 for each group). Error bars are SEM, asterisks denote p&lt;0.05 by Student’s t-test. Lateral views, anterior right (A-D). A, apical; B, basal. Scale bar: 50 mm.
(TIF)

S8 Fig Restoring FGF signaling does not restore ventricular integrity. (A) Graph depicting percentage of embryos with ectopic cardiomyocytes (Cyp26-deficient n = 49, Cyp26-deficient *Tg(hsp70*:*ca-fgfr1)* n = 41). (B) Graph depicting average number of ectopic cardiomyocytes in embryos with ectopic cardiomyocytes (Cyp26-deficient n = 14, Cyp26-deficient *Tg(hsp70*:*ca-fgfr1)* n = 9). (C) Graph depicting ventricular cell counts at 72 hpf of *Tg(hsp70*:*ca-fgfr1)* after a second heatshock at 48hpf. Error bars are SEM, asterisks denote p&lt;0.05 by Student’s t-test.
(TIF)

S9 Fig Increased *mmp9* expression in Cyp26-deficient embryos is not due to an increase in macrophage and neutrophil numbers. (A,B) ISH of *mmp9* in control and Cyp26-deficient embryos at 48 hpf. (C) RT-qPCR of *mmp9* expression in control, Cyp26c1-depleted, *gir* mutant, and *gir+c1* embryos. (D,E) ISH of *mfap4* in control and Cyp26-deficient embryos at 48 hpf. (F) Graph depicting quantification of average macrophage number over the yolk sac (n = 15 per group). (G,H) ISH of *mpx* in control and Cyp26-deficient at 48 hpf. (I) Graph depicting the average number of neutrophils over the yolk sac (n = 10 per group). Error bars are SEM. Lateral view, anterior left (A,B), frontal view, anterior up (D,E,G,H); n&gt;20 embryos per group (A,B,D,E,G,H). Scale bars: 50 mm.
(TIF)

S10 Fig GM6001 treatment restore ventricular cardiomyocyte polarity and shape in Cyp26-deficient embryos. (A-D) Confocal images of IHC of control (DMSO treated), GM6001 treated, Cyp26-deficient DMSO treated, Cyp26-deficient GM6001 treated *Tg(myl7*:*EGFP)* embryos stained for ZO1 (red) and GFP (green). (A’-D’) Schematized outlines of cell boundaries and ZO1 staining for images in A-D. (E) Graph depicting the percentage of ZO1 expression along the height of cardiomyocytes. (F) Graph depicting circularity measurement of ventricular cells. For E and F, control n = 18, GM6001 treated n = 15, Cyp26-deficient n = 21, Cyp26-deficient + GM6001 treated n = 21. Error bars are SEM, asterisks denote p&lt;0.05 by Student’s t-test compared to controls. Lateral views, anterior right (A-D). A, apical; B, basal. Scale bars: 25 mm.
(TIF)

S1 Data Data spreadsheet—Figures. Excel spreadsheet containing in separate sheets all the numerical data and statistical analysis for the Figure panels 1G, 1H, 1K, 2N, 2Q, 3G, 3J, 3K, 4A, 4B, 4C, 4D, 5A, 5B, 5C, 5D, 5M, 5N, 5Q, 5R, 5S, and 5T.
(XLSX)

S2 Data Data spreadsheet—Supporting Information. Excel spreadsheet containing in separate sheets all the numerical data and statistical analysis for the Figure panels S2Q, S2R, S2S, S2T, S3C, S3F, S3K, S3L, S7E, S7F, S8A, S8B, S8C, S9C, S9F, S10E, and S10F.
(XLSX)

S1 Movie Time-lapse movie of a depth coded, control *Tg(myl7*:*EGFP)* heart over the course of 12 hours. (MOV)

S2 Movie Time-lapse movie of a depth coded, Cyp26-deficient *Tg(myl7*:*EGFP)* heart over the course of 12 hours. (MOV)

## Abbreviations

aCasp3: activated Caspase 3
amhc: atrial myosin heavy chain
CHDs: congenital heart defects
Cyp: pan cytochrome P450
elnb: elastin b
FGF: fibroblast growth factor
fgf8a: fibroblast growth factor 8a
FHF: first heart field
gir: Giraffe
HE: hematoxylin–eosin
hpf: hours post fertilization
IHC: immunohistochemistry
ISH: in situ hybridization
MMP: matrix metalloproteinase
mmp9: matrix metalloproteinase 9
MOs: morpholinos
OFT: outflow tract
PAAs: pharyngeal arch arteries
PRC2: Polycomb repressive complex 2
RA: retinoic acid
RT-qPCR: real-time quantitative polymerase chain reaction
SHF: second heart field
ZO1: Zonula occludens 1
