# Supplementary figures and images for: Cyp26 Enzymes Facilitate Second Heart Field Progenitor Addition and Maintenance of Ventricular Integrity
Source: PLoS Biol. 2016 Nov 28;14(11):e2000504. doi: 10.1371/journal.pbio.2000504 (PMC5125711; doi:10.1371/journal.pbio.2000504)

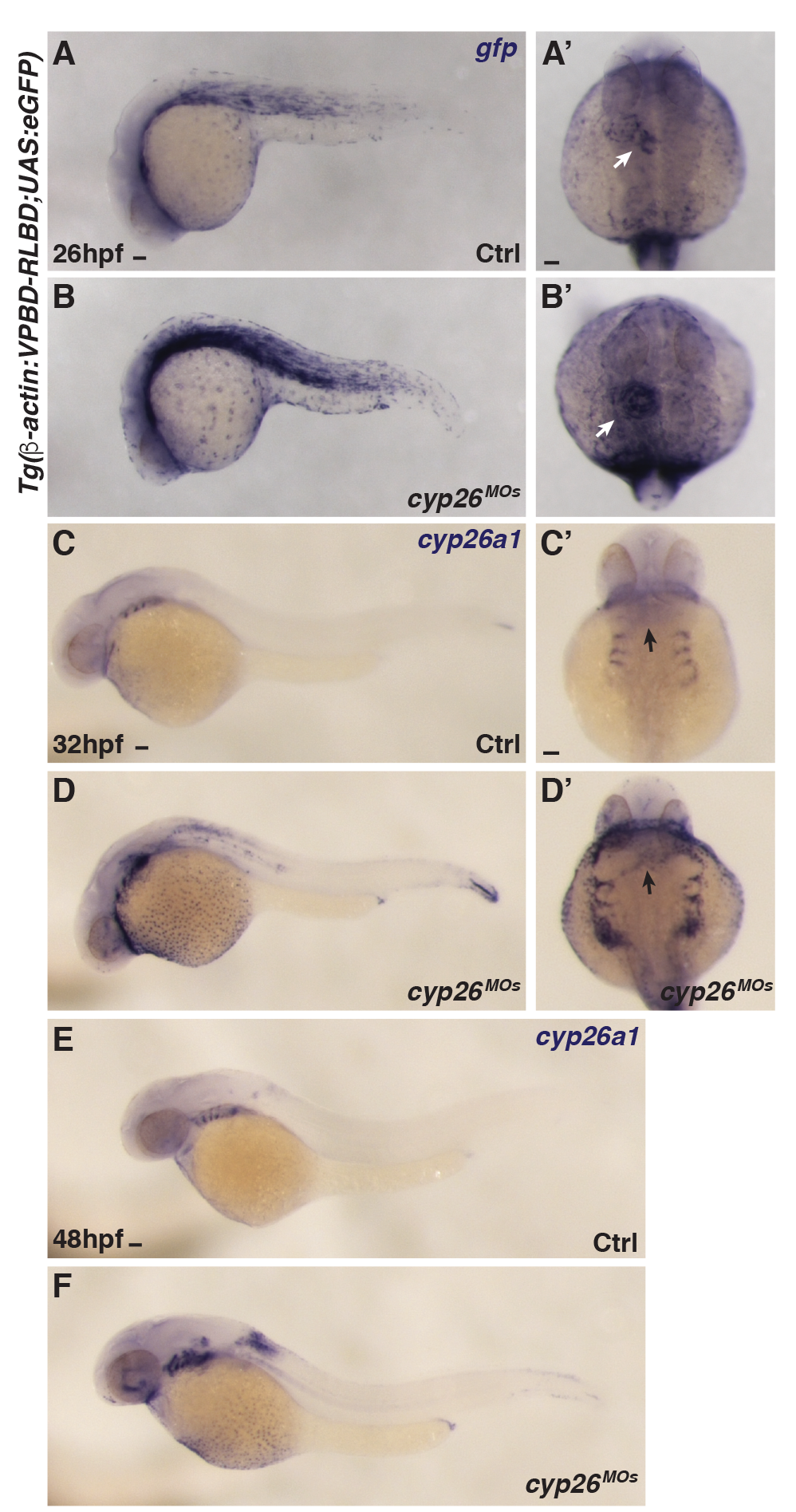

Supplement: S1 Fig — (A-B') ISH for gfp in WT and Cyp26-deficient Tg(β-actin:VPBD-RLBD;UAS:EGFP) embryos. Arrows in A’ and B’ indicate expression in the heart. (C-F) ISH for cyp26a1 in WT and Cyp26-deficient embryos at 32 and 48 hpf. Arrows in C’ and C’ indicate increased expression in the cardiac region. (A,B,C,D,E,F) Lateral view, anterior left, (A’,B’,C’,D’) Dorsal view, anterior up; n>20 embryos (A-F). Scale bars: 50 mm. (TIF) [file pbio.2000504.s001.tif]

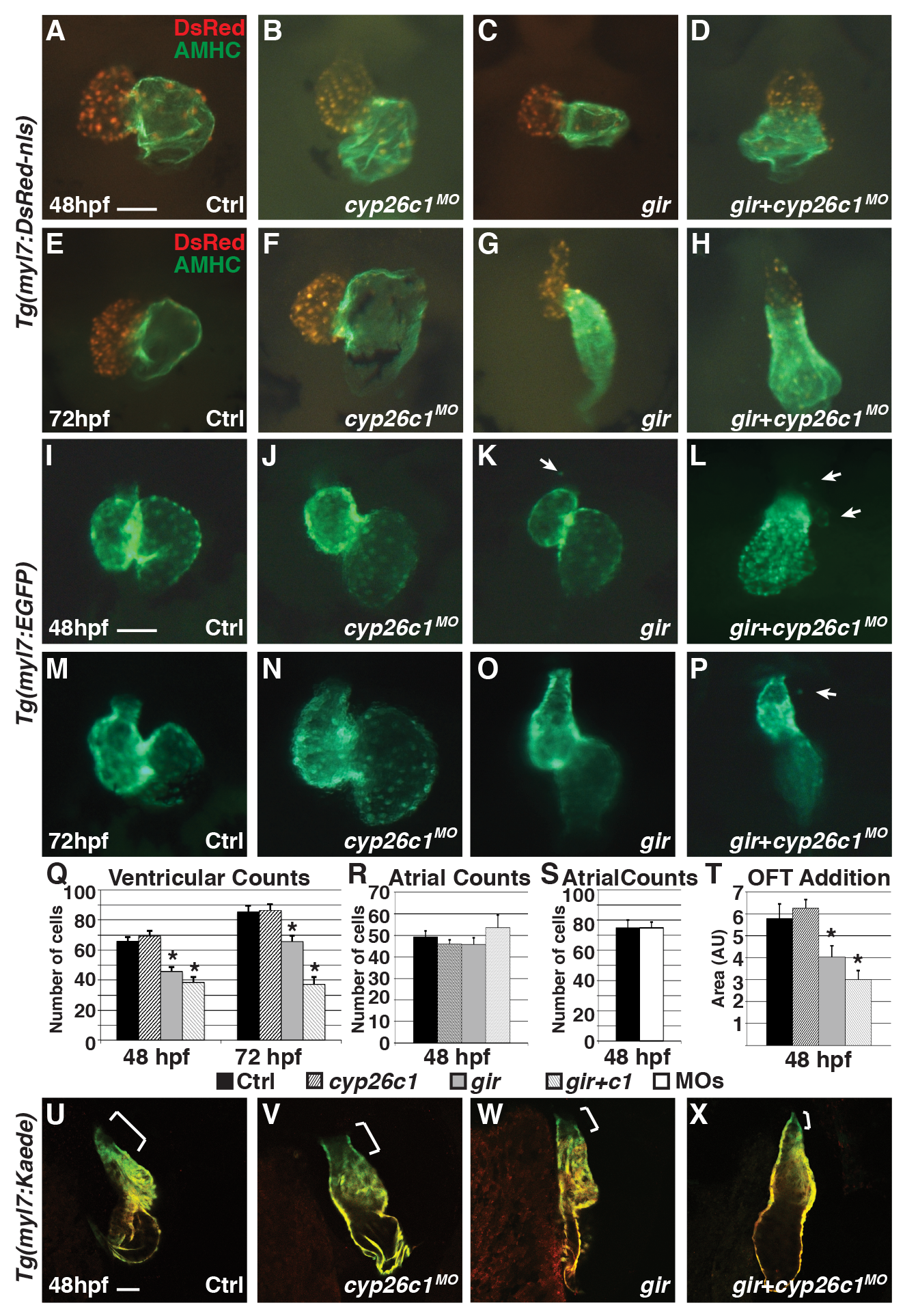

Supplement: S2 Fig — (A-H) IHC of hearts from control, Cyp26c1-depleted, gir mutant, and gir+c1 Tg(myl7:DsRed-NLS) embryos at 48 and 72 hpf. (I-P) Hearts from control, Cyp26c1-depleted, gir mutant, and gir+c1 Tg(myl7:EGFP) embryos at 48 and 72 hpf. Arrows denote ectopic cardiomyocytes. Ectopic cardiomyocytes were observed in 0/74 control, 1/80 Cyp26c1-depleted, 8/29 gir, and 7/31 gir+c1 at 48 hpf. (Q) Graph depicting ventricular cardiomyocyte counts at 48 and 72 hpf (n = 10 per group). (R) Graph depicting atrial cardiomyocyte counts at 48 hpf (n = 10 per group). (S) Graph depicting atrial cardiomyocyte counts at 48 hpf (n = 10 per group) in control and Cyp26-deficient embryos. (T) Graph of ventricular addition to the OFT (n = 10 per group). (U-X) Control, cyp26c1 MO injected, gir, and gir+c1 Tg(myl7:Kaede) embryo hearts at 48 hpf after photoconversion at 36 hpf. Brackets indicate ventricular addition (green only). Error bars are SEM, asterisks denote p<0.05 compared to controls by Student’s t-test. All images are frontal views with anterior up; n>20 embryos for (U-X). Scale bars: 50 mm. (TIF) [file pbio.2000504.s002.tif]

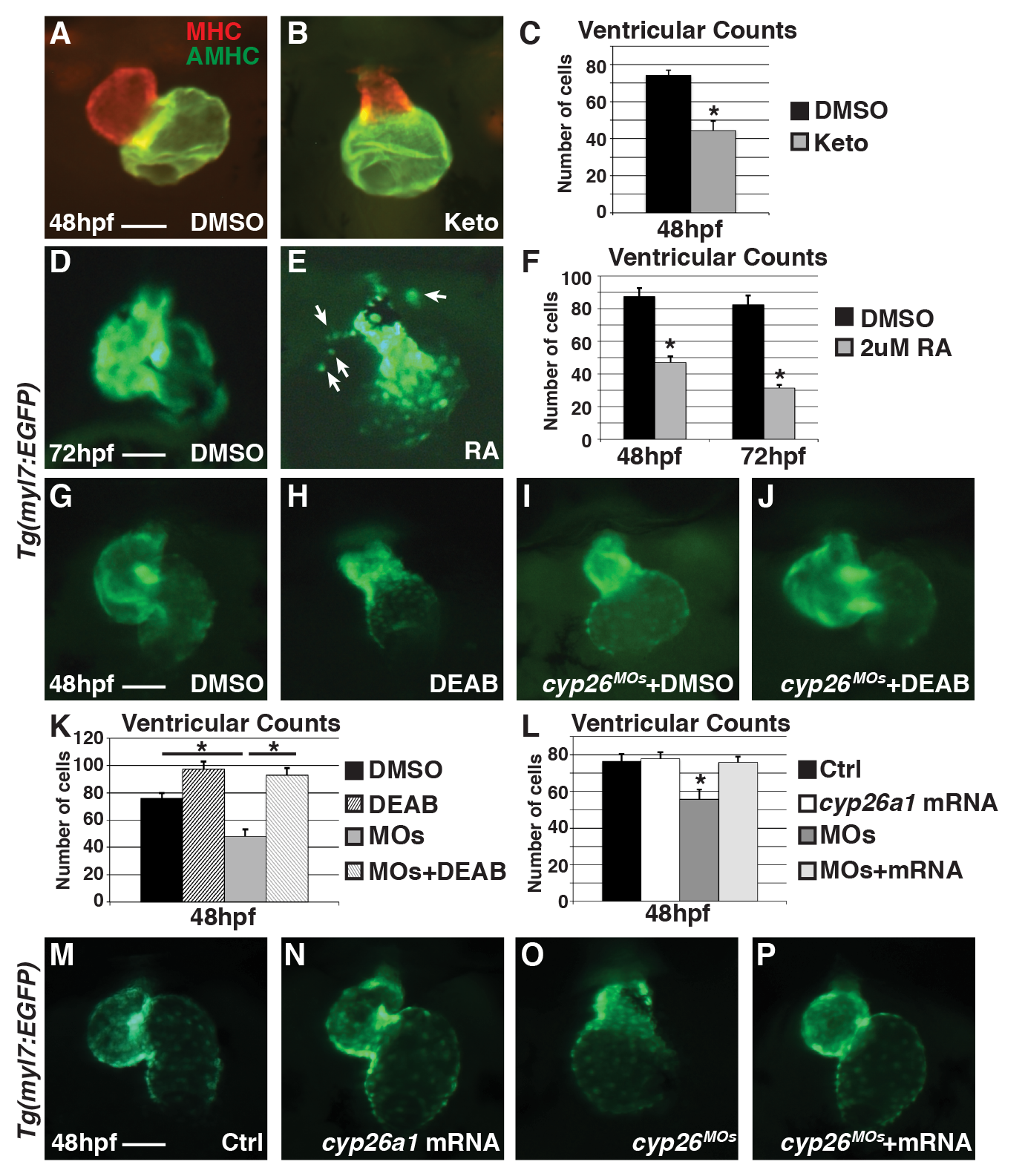

Supplement: S3 Fig — (A,B) IHC of DMSO or Ketoconazole (Keto) treated embryos at 48 hpf with ventricle in red (MHC) and atrium in green (AMHC). (C) Graph depicting ventricular counts of DMSO or Ketoconazole treated embryos at 48 hpf. (D,E) Hearts of Tg(myl7:EGFP) embryos at 72 hpf that were treated with DMSO or 2 μM RA beginning at 24 hpf. Arrows denote ectopic cardiomyocytes. (F) Graph of ventricular counts embryos at 48 and 72 hpf (n = 10 per group) of DMSO or 2 μM RA treatment beginning at 24 hpf. (G-J) Hearts at 48 hpf in control or Cyp26-deficient Tg(myl7:EGFP) embryos treated with DMSO or DEAB. (K) Graph depicting ventricular cell counts at 48 hpf for DMSO, DEAB, Cyp26-deficient + DMSO, and Cyp26-deficient + DEAB treated embryos. (L) Graph depicting ventricular cell counts at 48 hpf in control and Cyp26-deficient Tg(myl7:DsRed-NLS) embryos injected with cyp26a1 mRNA. (M-P) Hearts at 48 hpf from control, cyp26a1 mRNA-injected, Cyp26-deficient and Cyp26-deficient injected with cyp26a1 mRNA Tg(myl7:EGFP) embryos. Error bars are SEM, asterisks denote p<0.05 by Student’s t-test. All images are frontal views with anterior up; n>20 embryos for (A,B,D,E,G-J,M-P). Scale bars: 50 mm. (TIF) [file pbio.2000504.s003.tif]

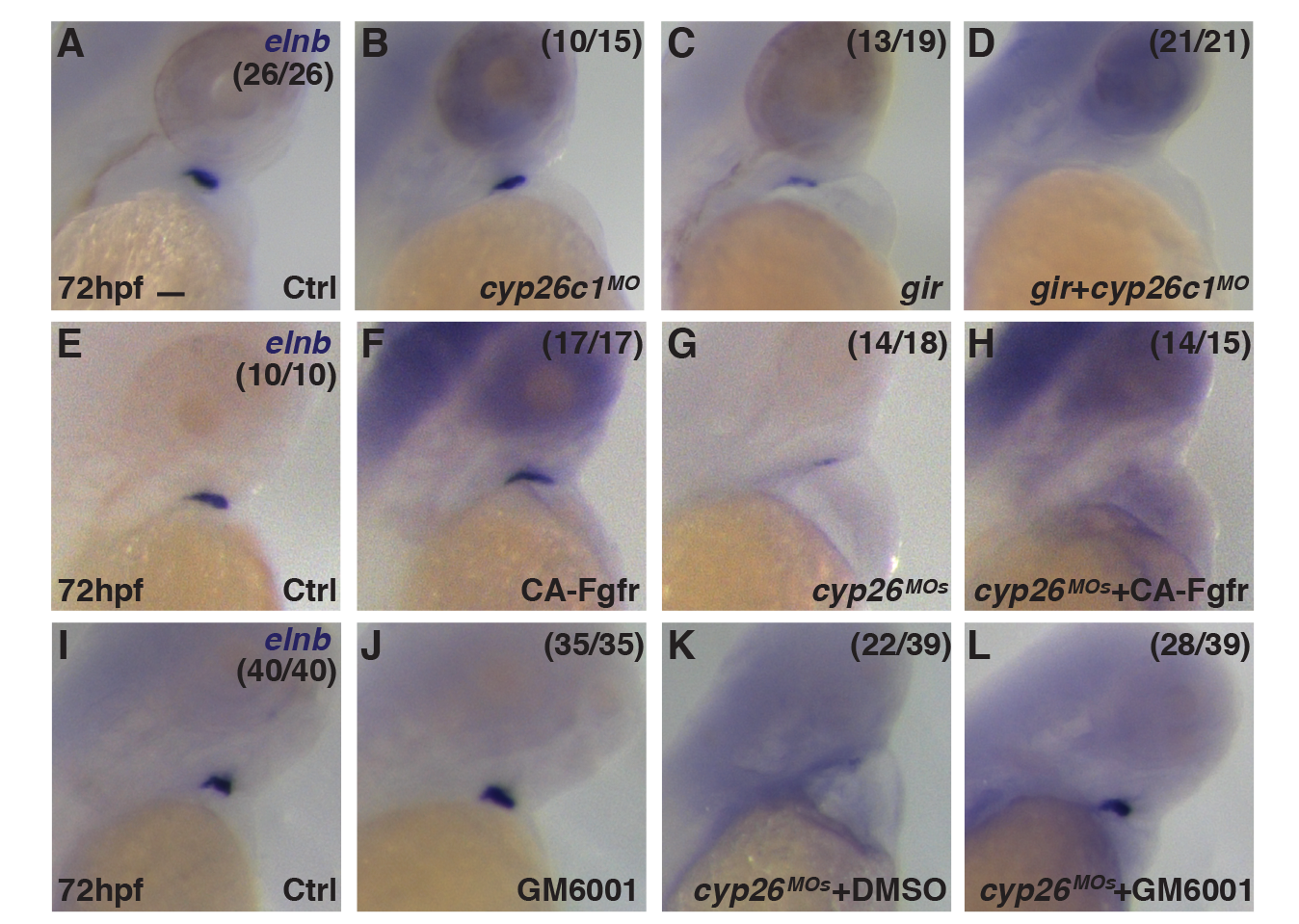

Supplement: S4 Fig — (A-D) ISH for elnb in control, Cyp26c1-depleted, gir mutant, and gir+c1 embryos. (E-H) ISH for elnb in control and Cyp26-deficient embryos after heat-shock induction of FGF. (I-L) ISH for elnb in control or Cyp26-deficient embryos treated with DMSO or GM6001.Control embryo in I were treated with DMSO. Lateral view, anterior up (A-L). Scale bar: 50 mm. (TIF) [file pbio.2000504.s004.tif]

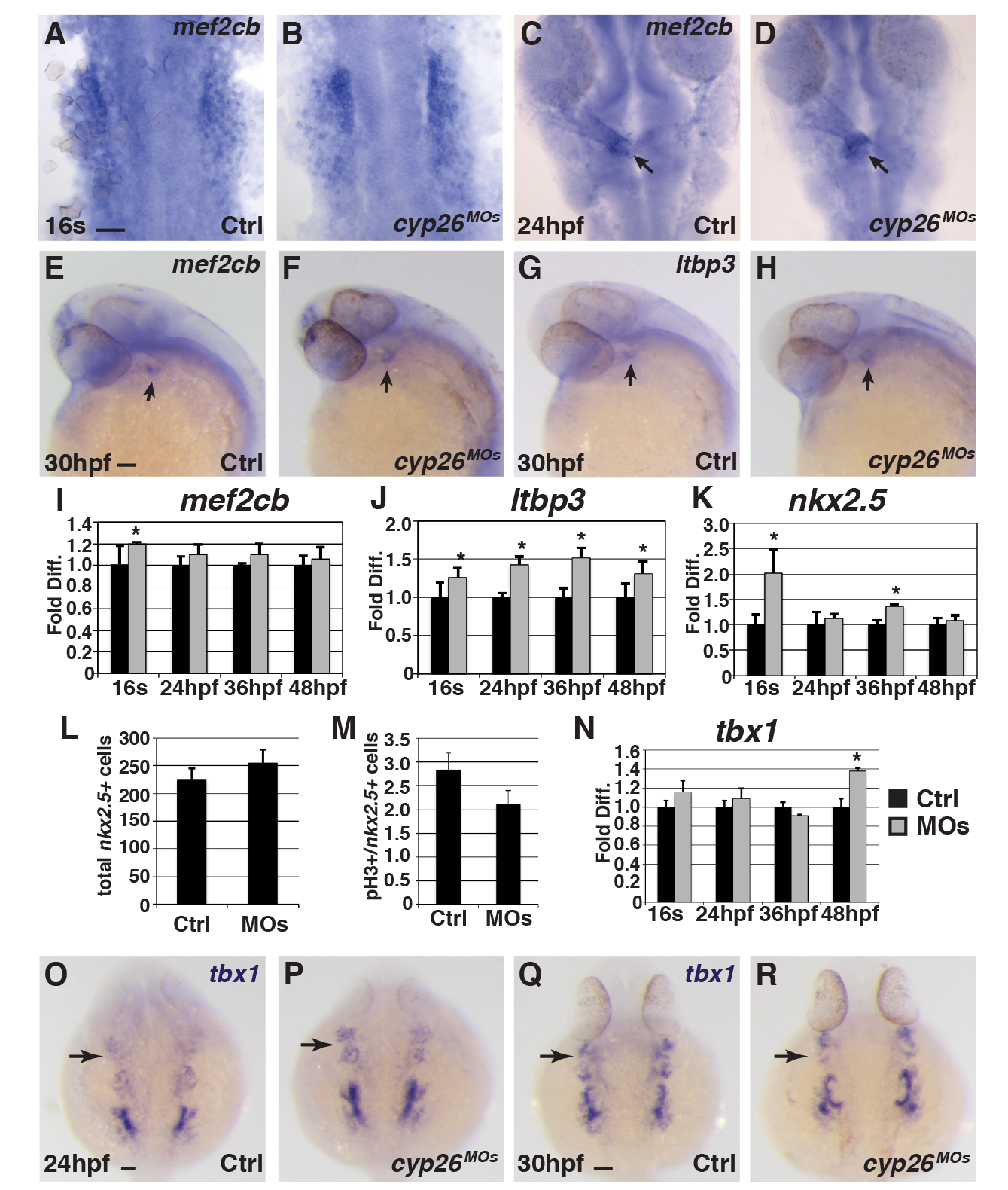

Supplement: S5 Fig — (A-F) ISH of mef2cb at 16 somites (s), 24, and 30 hpf in control and Cyp26-deficient embryos. (G,H) ISH of ltbp3 at 30 hpf in control and Cyp26-deficient embryos. Arrows in C-H indicate expression in the OFT. (I) RT-qPCR for mef2cb at 16 s, 24, 36 and 48 hpf. (J) RT-qPCR for ltbp3 at 16 s, 24, 36 and 48 hpf. (K) RT-qPCR for nkx2.5 at 16 s, 24, 36 and 48 hpf. (L) Graph depicting total number of nkx2.5+ cells at 24 hpf (n = 11 for control, n = 14 for Cyp26-deficient). (M) Graph depicting the percentage of proliferating nkx2.5+ cells at 24 hpf (n = 11 for control, n = 14 for Cyp26-deficient). (N) RT-qPCR for tbx1 at 16 s, 24, 36, and 48 hpf. For RT-qPCR, fold difference of mRNA was calculated relative to β-actin. (O-R) ISH for tbx1 at 24 and 30 hpf in control and Cyp26-deficient embryos. Arrows indicate tbx1 staining adjacent to the heart. Error bars are SEM, asterisks denote p<0.05 by Student’s t-test. Dorsal view, anterior up (A-D), lateral view anterior right (E-H); n>20 embryos per group for (A-H). Scale bars: 50 mm. (TIF) [file pbio.2000504.s005.tif]

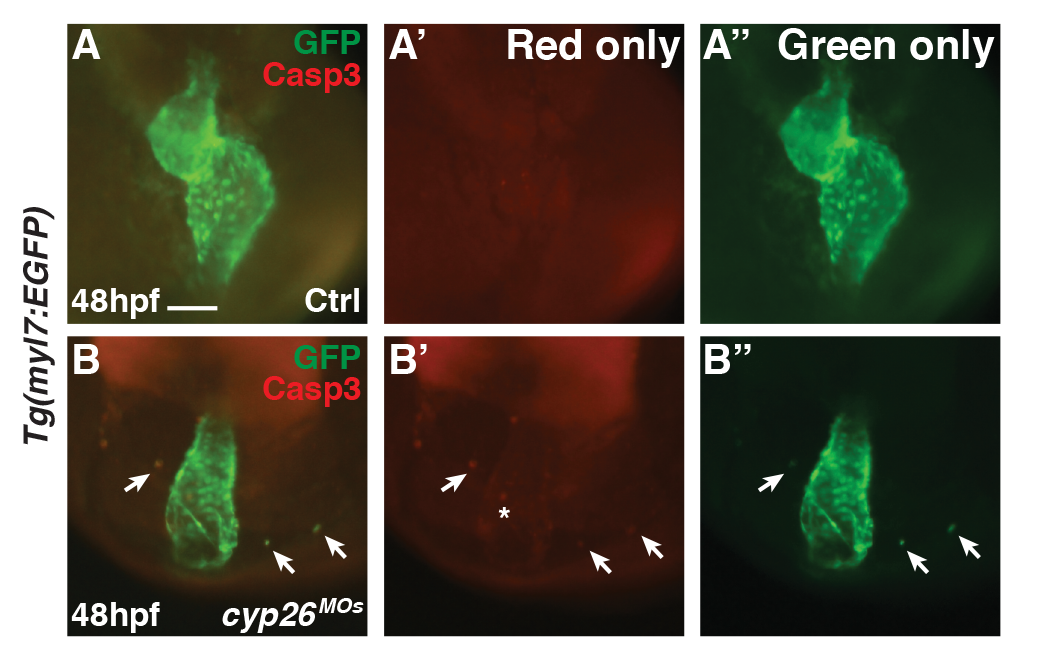

Supplement: S6 Fig — (A,B) IHC of control or Cyp26-deficient Tg(myl7:EGFP) embryos for aCasp3 (red) and GFP (green). Arrows denote ectopic cardiomyocytes co-expressing aCasp3. 16/18 ectopic cardiomyocytes were co-labeled. Asterisk denotes aCasp3+ cell potentially within the heart tube. Frontal view, anterior up; Control n = 30. Cyp26-deficient n = 11 embryos. Scale bar: 50 mm. (TIF) [file pbio.2000504.s006.tif]

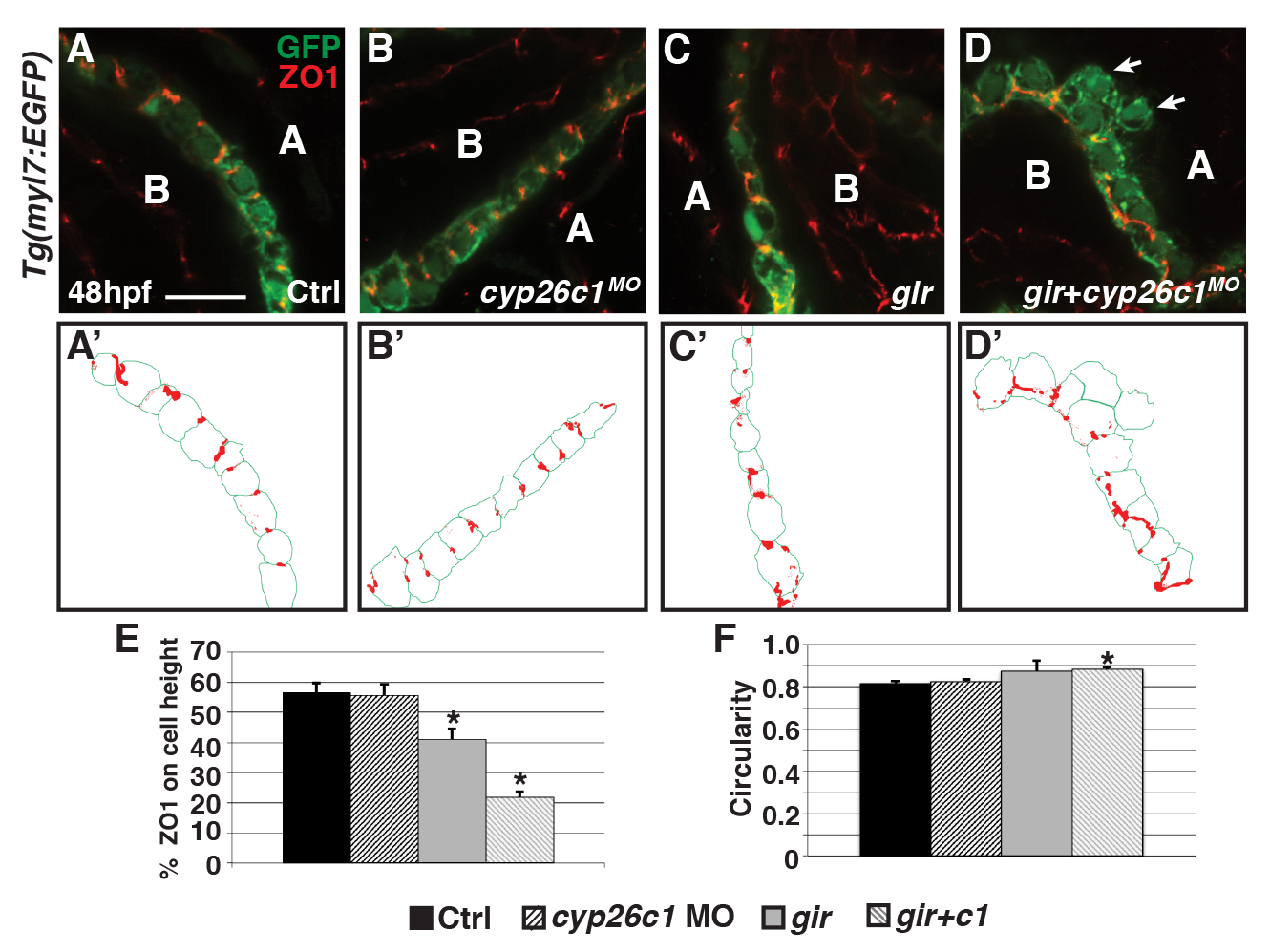

Supplement: S7 Fig — (A-D) Confocal images of IHC of control, Cyp26c1-depleted, gir mutant, and gir+c1 Tg(myl7:EGFP) stained for ZO1 (red), and GFP (green). (A’-D’) Schematized outlines of cell boundaries and ZO1 staining for images in A-D. Arrows denotes cardiomyocytes protruding into the pericardial space. (E) Graph depicting the percentage of ZO1 expression along the height of cardiomyocytes (n = 36 for each group). (F) Graph depicting circularity measurement of ventricular cells (n = 36 for each group). Error bars are SEM, asterisks denote p<0.05 by Student’s t-test. Lateral views, anterior right (A-D). A, apical; B, basal. Scale bar: 50 mm. (TIF) [file pbio.2000504.s007.tif]

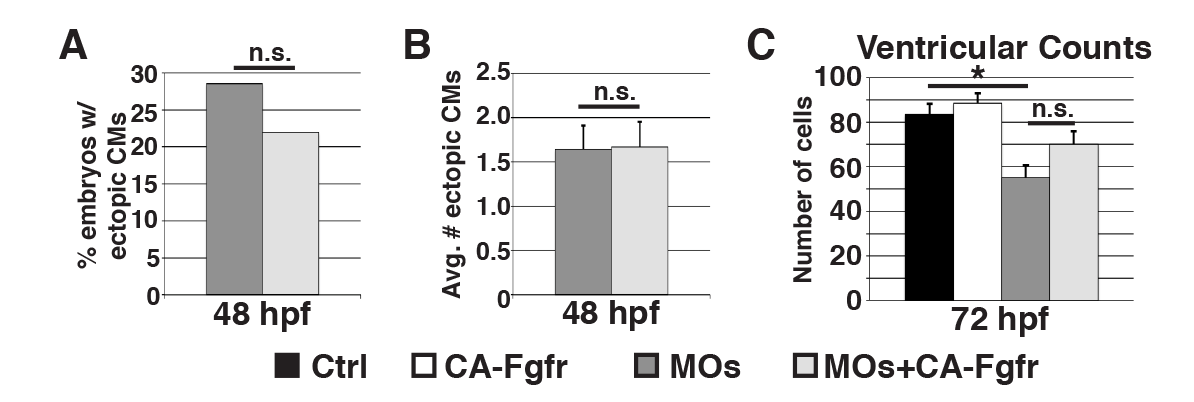

Supplement: S8 Fig — (A) Graph depicting percentage of embryos with ectopic cardiomyocytes (Cyp26-deficient n = 49, Cyp26-deficient Tg(hsp70:ca-fgfr1) n = 41). (B) Graph depicting average number of ectopic cardiomyocytes in embryos with ectopic cardiomyocytes (Cyp26-deficient n = 14, Cyp26-deficient Tg(hsp70:ca-fgfr1) n = 9). (C) Graph depicting ventricular cell counts at 72 hpf of Tg(hsp70:ca-fgfr1) after a second heatshock at 48hpf. Error bars are SEM, asterisks denote p<0.05 by Student’s t-test. (TIF) [file pbio.2000504.s008.tif]

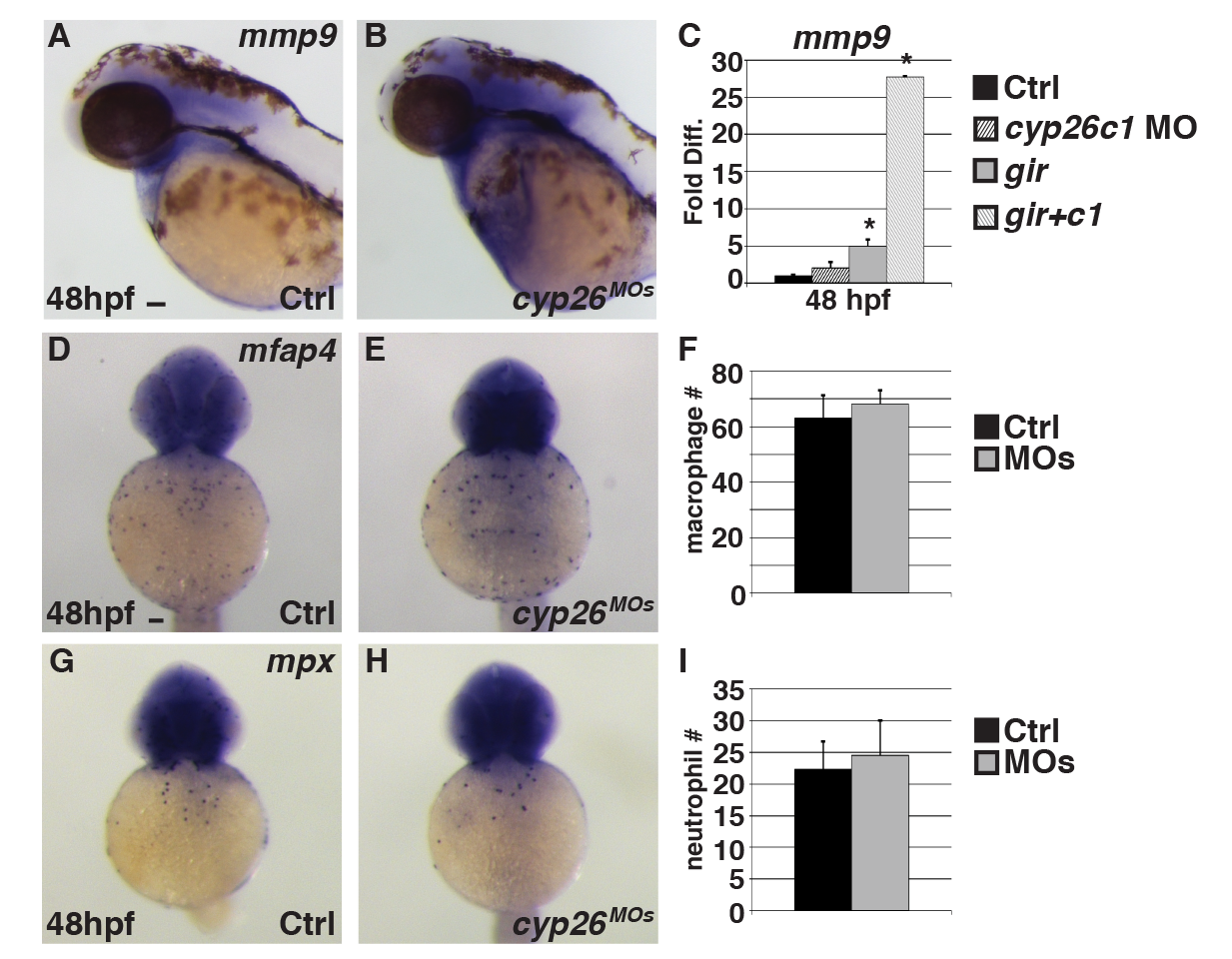

Supplement: S9 Fig — (A,B) ISH of mmp9 in control and Cyp26-deficient embryos at 48 hpf. (C) RT-qPCR of mmp9 expression in control, Cyp26c1-depleted, gir mutant, and gir+c1 embryos. (D,E) ISH of mfap4 in control and Cyp26-deficient embryos at 48 hpf. (F) Graph depicting quantification of average macrophage number over the yolk sac (n = 15 per group). (G,H) ISH of mpx in control and Cyp26-deficient at 48 hpf. (I) Graph depicting the average number of neutrophils over the yolk sac (n = 10 per group). Error bars are SEM. Lateral view, anterior left (A,B), frontal view, anterior up (D,E,G,H); n>20 embryos per group (A,B,D,E,G,H). Scale bars: 50 mm. (TIF) [file pbio.2000504.s009.tif]

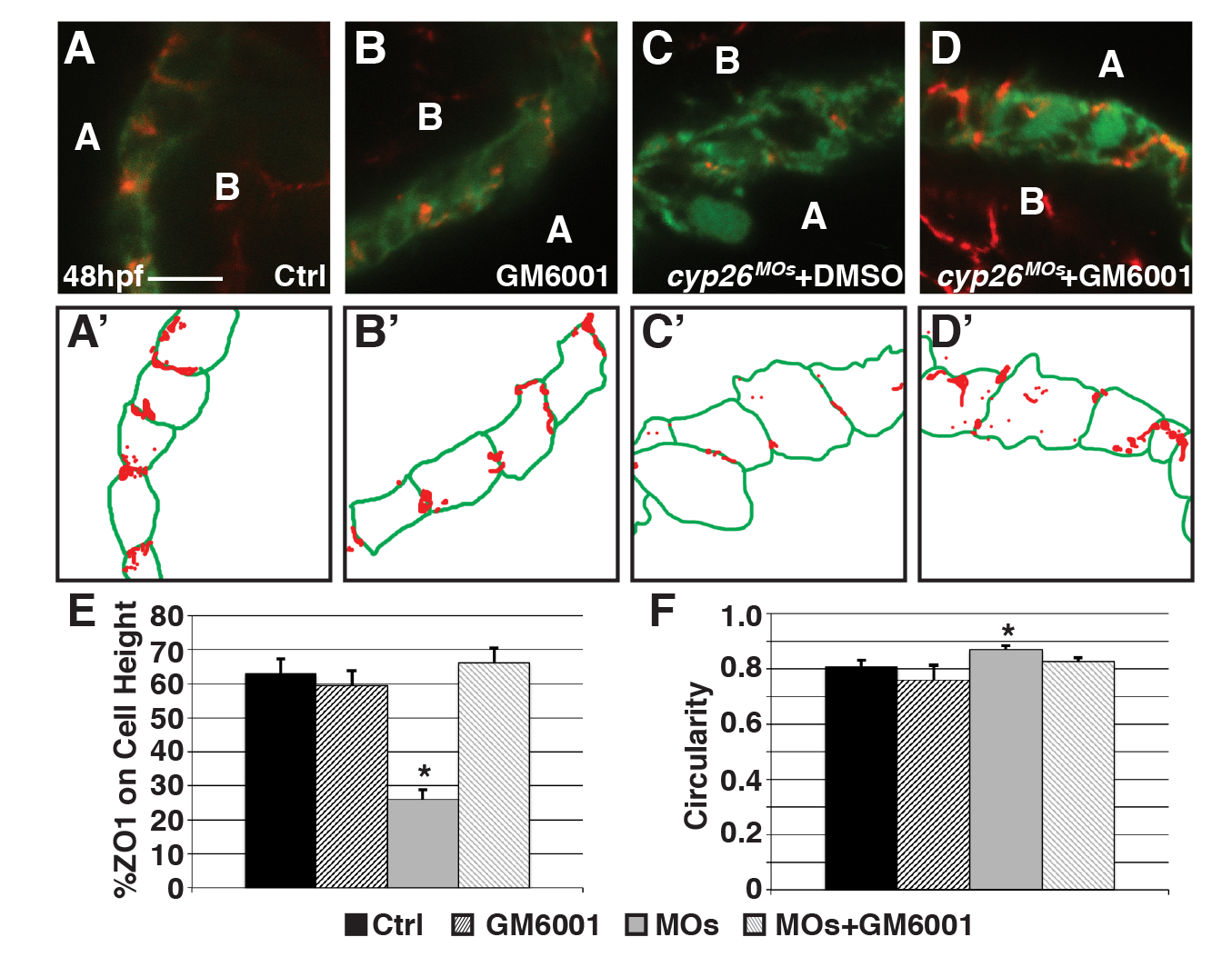

Supplement: S10 Fig — (A-D) Confocal images of IHC of control (DMSO treated), GM6001 treated, Cyp26-deficient DMSO treated, Cyp26-deficient GM6001 treated Tg(myl7:EGFP) embryos stained for ZO1 (red) and GFP (green). (A’-D’) Schematized outlines of cell boundaries and ZO1 staining for images in A-D. (E) Graph depicting the percentage of ZO1 expression along the height of cardiomyocytes. (F) Graph depicting circularity measurement of ventricular cells. For E and F, control n = 18, GM6001 treated n = 15, Cyp26-deficient n = 21, Cyp26-deficient + GM6001 treated n = 21. Error bars are SEM, asterisks denote p<0.05 by Student’s t-test compared to controls. Lateral views, anterior right (A-D). A, apical; B, basal. Scale bars: 25 mm. (TIF) [file pbio.2000504.s010.tif]
